# Ginger Bioactives as Multi-Target Therapeutics: Mechanisms, Delivery Innovation, and Human Health Impact

**DOI:** 10.3390/nu18071079

**Published:** 2026-03-27

**Authors:** Pasquale Simeone, Francesca Martina Filannino, Antonia Cianciulli, Maria Ida de Stefano, Melania Ruggiero, Teresa Trotta, Antonella Compierchio, Tarek Benameur, Rosa Calvello, Amal Ferchichi, Chiara Porro, Maria Antonietta Panaro

**Affiliations:** 1 Department of Clinical and Experimental Medicine, University of Foggia, 71122 Foggia, Italy; pasquale.simeone@unifg.it (P.S.); francesca.filannino@unifg.it (F.M.F.); maria.destefano@unifg.it (M.I.d.S.); teresa.trotta@unifg.it (T.T.); 2 Department of Biosciences, Biotechnologies and Environment, University of Bari, 70125 Bari, Italy; antonia.cianciulli@uniba.it (A.C.); melania.ruggiero@uniba.it (M.R.); a.compierchio2@phd.uniba.it (A.C.); rosa.calvello@uniba.it (R.C.); 3 Department of Biomedical Sciences, College of Medicine, King Faisal University, Al-Ahsa 31982, Saudi Arabia; 4 Laboratory of Mycology, Pathologies, and Biomarkers (LR16/ES05), Department of Biology, Faculty of Sciences of Tunis, University of Tunis El Manar, Tunis 2092, Tunisia; ferchichiamal0708@gmail.com

**Keywords:** ginger, *Zingiber officinale* Roscoe, gingerol, shogaol, bioactive compounds, anti-inflammatory, anti-apoptotic, nanotechnology, nutraceutical, artificial intelligence, health

## Abstract

**Background/Objectives**: Ginger has a long history as both a culinary and medicinal plant and is widely recognized in traditional medicine for its ability to promote health and well-being. The principal bioactive compounds of ginger are present in fresh and dried forms and have been largely studied for their therapeutic potential. These compounds exhibit a wide range of biological activities mediated through various mechanisms. Advances in nanotechnology have enabled the development of innovative delivery systems, thereby enhancing the bioavailability and therapeutic efficacy of ginger-derived compounds in modern medical applications. **Methods**: A comprehensive literature review was conducted to evaluate the characteristics of ginger and its potential role in disease prevention. Relevant studies were identified through the main research databases, publication screening, manual reference checks, and author consensus was conducted. **Results**: This narrative review provides an overview of the therapeutic potential of bioactive compounds in ginger for the management and prevention of cardiovascular, arthritis, neurodegenerative, and gastrointestinal diseases, with particular emphasis on the molecular mechanisms. In addition, their potential anti-aging properties are extensively discussed. The evidence reported is predominantly preclinical (*in vitro* and *in vivo* models), with more limited and heterogeneous clinical data. Recent studies have also highlighted the role of artificial intelligence (AI) in accelerating the discovery and evaluation of bioactive agents with therapeutic relevance across diverse biological systems. **Conclusions**: This review highlights the emerging applications of ginger extracts in human health and suggests their applications in both traditional medicine and contemporary drug discovery.

## 1. Introduction and Background

Ginger (*Zingiber officinale* Roscoe) is a perennial herb belonging to the Zingiberaceae family and is predominantly found in Southeast Asia [1,2]. The Zingiberaceae family contains approximately 47 genera and more than 1000 species, characterized by considerable diversity in floral morphology and rhizome structure [3,4,5].

The precise geographic origin of ginger remains uncertain; however, historical evidence suggests that it was first cultivated in China and neighboring regions of Southeast Asia [6]. In the 13th century, ginger has been introduced to Europe, while Arab traders and later the Portuguese facilitated its spread to East and West Africa, respectively [7].

Ginger is now widely cultivated and distributed worldwide, including in China, India, Saudi Arabia, Nigeria, Indonesia, Japan, Burma, Taiwan, Australia, Sri Lanka, Germany, Greece, and many other countries [8]. Ginger thrives in warm climates and is highly sensitive to very low temperatures, making tropical and subtropical climates optimal for its cultivation.

Notably, the composition of its bioactive compounds varies with growing conditions, geographical location, and post-harvest drying methods [9,10]. The harvesting period depends on the intended end use. For products such as preserves, confectionery, beverages, or fresh consumption, ginger is typically harvested 4–5 months after planting. In contrast, when used for dried, blanched, or dehydrated products, or for the extraction of functional constituents such as essential oil and oleoresin, harvesting is generally extended to approximately 8–10 months [9].

The ginger plant is an erect herbaceous species, forming a pseudostem reaching approximately 30 cm to 1 min height. Its rhizome is irregularly branched, coarsely fibrous, horizontally oriented, and internally pale yellow in color. Roots emerge from the lower surface of the rhizome and can remain viable in the soil for extended periods, facilitating the production of new shoots [5].

The U.S. Food and Drug Administration (FDA) classifies ginger root as a generally safe herbal supplement for use in complementary and alternative medicine formulations [11]. Ginger is also extensively used as a culinary spice due to its characteristic refreshing aroma and spicy, pungent taste [7]. The profile and concentration of ginger bioactive compounds are substantially affected by multiple factors, including maturity stage, climatic conditions, soil properties, and post-harvest processing parameters. In addition, preservation techniques, thermal treatments, and extraction methods further influence the chemical composition and functional properties of ginger-derived products [12].

Ginger rhizomes contain carbohydrates, proteins, ash, crude fibers, lipids, water, and essential mineral elements, including sodium, potassium, calcium, magnesium, and phosphorus [13]. In addition to these macronutrients, ginger is rich in both phenolic compounds (non-volatile constituents) and terpenoid components (volatile compounds), many of which possess significant pharmacological properties. The terpene composition can vary considerably depending on the geographic origin of the plant [14].

Together with shogaols, these compounds represent the principal bioactive compounds of ginger, and their concentrations differ markedly between fresh and dried ginger due to thermal processing. In particular, gingerols are the predominant constituents in fresh ginger, whereas shogaols dominate in dried ginger because heat exposure and drying promote the conversion of gingerols to shogaols, thereby increasing their abundance [15,16]. These polyphenols exhibit several biological activities, including antioxidant, anti-inflammatory, antitumoral, and antidiabetic activities [11]. 6-gingerol is the main bioactive compound present in fresh, non-processed ginger rhizome, but dehydration processes promote its transformation into 6-shogaol [15].

Throughout history, ginger rhizomes have been utilized not only as a spice and flavoring agent in foods and beverages but also for various medicinal purposes [17,18]. Traditionally, ginger has been largely used in the management of various ailments, including arthritis, rheumatism, sprains, muscular pain, headache, toothache, cramps, diarrhea, constipation, gastrointestinal discomfort, indigestion, nausea, vomiting, hypertension, dementia, fever, infectious diseases, helminthiasis, respiratory disorders, rheumatic disorders, and other related disorders [19,20,21].

In recent years, extensive research and documented scientific evidence have confirmed the pharmacological potential of ginger, demonstrating a broad spectrum of bioactivities, including anticancer, antimicrobial, immunomodulatory, antiapoptotic, antihyperglycemic, antilipidemic, and antiemetic effects [22,23].

These findings, together with the increasing interest in natural products characterized by relatively low toxicity, suggest that ginger-derived compounds may provide substantial health benefits. Consequently, this has prompted extensive research to further investigate ginger’s bioactive compounds and to evaluate their therapeutic potential in the context of human health.

Based on these considerations, this narrative review aims to provide a comprehensive overview of the therapeutic potential of ginger-derived bioactive compounds in different pathological conditions, including cardiovascular, neurodegenerative, arthritic, and gastrointestinal diseases, with particular emphasis on the underlying molecular and mechanistic pathways. The anti-aging properties of ginger compounds are also discussed in detail. Furthermore, recent advances in Artificial Intelligence (AI) have demonstrated its utility in accelerating the discovery of biologically active therapeutic agents across diverse complex biological systems. In this context, the present review analyses, through integrative approaches, the emerging applications of ginger-derived compounds in human health, supporting evidence-based applications in both traditional medicine and modern drug discovery in order to prevent important pathologies and to improve quality of life.

## 2. Methodological Approach

In this narrative review, we first described the generic characteristics of ginger and then explored its potential role in the prevention of various pathological conditions. The literature search was conducted collaboratively by multiple authors using PubMed database and was limited to articles published in English. Boolean operators (AND, OR, NOT) were applied to combinations of relevant keywords and phrases, including “ginger”, “*Zingiber officinale*”, “gingerol”, “shogaol”, along with other terms such as “clinical trial”, “randomized”, “human”, “health”. To enhance the comprehensiveness of the review, additional databases such as Scopus, Web of Science, and Embase could also be consulted. This electronic search was complemented by a manual screening of reference lists from selected articles and by critical discussion among all authors to ensure comprehensive coverage and relevance of the included studies.

## 3. Ginger Chemical Composition, Bioactive Compounds, and Their Properties

Ginger shows a complex chemical composition predominantly comprising phenolic constituents such as gingerols, shogaols, paradols, and zingerone. In fresh rhizomes, which typically contain 80–90% moisture, gingerols, particularly 6-gingerol, are the principal bioactive compounds and are primarily responsible for the characteristic pungency and therapeutic properties of the plant [4,15,24]. During drying, heat treatment, or prolonged storage, gingerols undergo dehydration of their thermally labile β-hydroxyketone moiety, resulting in the formation of shogaols, which are thermodynamically more stable and less susceptible to thermal degradation [15,25]. As reported by Ghasemzadeh A. et al., the rate of conversion increases with temperature, and the efficiency of transformation varies depending on the heating method employed. For example, extraction at 76.9 °C for 3.4 h yielded 2.89 mg/g DW of 6-gingerol and 1.85 mg/g DW of 6-shogaol. Moist heat treatment at 120 °C for 360 min produced significantly higher levels of shogaols compared with dry heat, reaching up to 2991 mg/kg of ginger [15,26].

Shogaols have been reported to show greater biological efficacy than gingerols [27], and enhanced pharmacological efficacy has been further documented in additional experimental models [28]. Therefore, the choice of drying method is critical, as it effectively transforms the phytochemical profile from a gingerol-rich fresh matrix to a shogaol-enriched product with modified biological activity.

In addition to phenolic constituents, ginger contains a diverse range of terpenoids, including monoterpenoids and sesquiterpenoids, such as zingiberene and zerumbone. These compounds contribute not only to the distinctive aroma and flavor of ginger but also to its pharmacological activities. Of particular interest, zerumbone, a sesquiterpene, has demonstrated an important anti-inflammatory and anticancer potential [29]. Its sesquiterpene scaffold allows it to modulate multiple disease-related pathways, including the suppression of inflammatory responses and the inhibition of tumor angiogenesis, making it a promising candidate for drug development [29]. In this respect, zerumbone is primarily derived from *Zingiber zerumbet*, a related species within the Zingiberaceae family, highlighting the broader bioactive potential of this botanical group [30].

In addition to zerumbone, other terpenoids present in ginger also contribute significantly to its antioxidant and antimicrobial activities [31]. Increasing evidence indicates that ginger has strong antibacterial properties, attributed to its chemically diverse composition. Studies have demonstrated its inhibitory effects against the growth of several bacterial strains, including *Escherichia coli*, *Staphylococcus aureus*, and *Salmonella enterica*, with gingerol, shogaol, and paradol identified as the principal compounds responsible for these effects [32,33].

Detailed phytochemical analyses further reveal that 6-gingerol is the most abundant and biologically active among the gingerols, while 10-gingerol and 6-shogaol also play significant roles. In particular, 6-shogaol has been extensively investigated for its potent anti-inflammatory, antioxidant, and antitumor activities [34].

**Figure 1:**
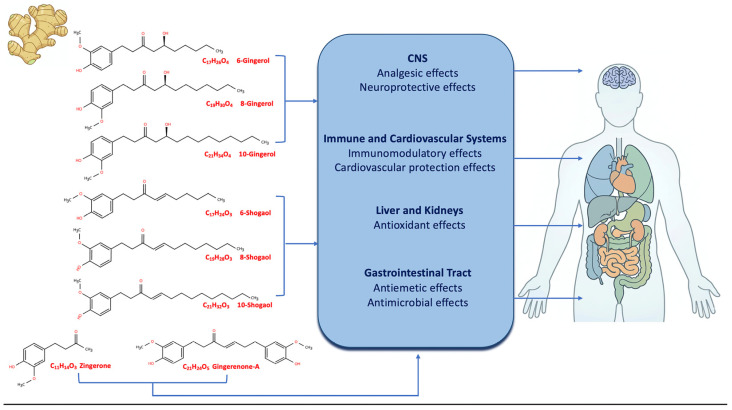
Bioactive compounds of ginger and their therapeutic effects on human health. The major constituents of ginger include (6-, 8-, and 10-gingerol), shogaols (6-, 8-, and 10-shogaol), zingerone, and gingerenone-A. These components exert analgesic and neuroprotective effects in the CNS, immunomodulatory and cardioprotective effects, antioxidant effects in the liver and kidneys, anti-antiemetic and antimicrobial effects in the gastrointestinal tract.

In addition to these major bioactive groups, ginger contains other classes of compounds, including polysaccharides, flavonoids, lipids, organic acids, and dietary fiber, which collectively enhance its nutritional value and contribute to its biological functions [35]. Importantly, these bioactive constituents often act synergistically, forming a complex phytochemical matrix that underlies ginger’s multiple health benefits, including anti-obesity, anti-diabetic, and cardioprotective effects [2,35] (Figure 1).

As shown in Figure 1, the main phenolic compounds of ginger, represented by gingerols, shogaols, paradols, and zingerone, share a phenolic hydroxyl group, which is crucial for antioxidant activity, while shogaols additionally contain an α, β unsaturated carbonyl, enhancing anti-inflammatory potential. Variations in the aliphatic side chain length and saturation influence lipophilicity, bioavailability, and organ-specific effects.

Other minor constituents, including flavonoids and various phenolic acids, further contribute to the overall antioxidant capacity and biological activity of ginger. Indeed, these compounds have been shown to modulate oxidative stress and inflammatory responses, key processes underlying the pathophysiology of numerous chronic diseases [36]. Collectively, the full spectrum of ginger’s bioactive molecules highlights its pleiotropic effects, manifesting in a wide range of cellular and physiological responses.

## 4. Antioxidant, Anti-Inflammatory, Antimicrobial, and Immunomodulatory Effects of Ginger

Among the most extensively studied therapeutic effects of ginger are its anti-inflammatory and antioxidant properties. These effects are largely attributed to its bioactive phenolic compounds, particularly gingerols and shogaols, which are considered the principal mediators of its biological activity. Their capacity to scavenge reactive oxygen species (ROS) and other free radicals plays a central role in mediating antioxidant and anti-inflammatory effects. Neutralizing ROS and free radicals can modulate the inflammatory signaling pathways, offering significant health benefits, especially in the context of chronic disease prevention.

In this context, ginger leaves have exhibited higher antioxidant activity and greater phenolic and flavonoid content than the rhizomes and stems, as measured by the 1,1-diphenyl-2-picryl-hydrazyl (DPPH) assay [37,38]. In addition to their direct radical-scavenging activity, ginger-derived phenolics have been shown to enhance endogenous antioxidant defense systems. Specifically, they can upregulate key antioxidant enzymes, including superoxide dismutase (SOD), catalase (CAT), and glutathione peroxidase (GPx). For example, extracts enriched in 6-shogaol have been reported to activate the Nrf2/antioxidant response element (ARE) signaling pathway and to modulate mitogen-activated protein kinase (MAPK) cascades, thereby enhancing cellular redox homeostasis and strengthening cytoprotective defense mechanisms [39].

Evidence from both *in vitro* and *in vivo* studies shows that ginger extract attenuates hepatic oxidative markers and cellular damage, contributing to the restoration of antioxidant balance [39,40].

In this regard, mice treated with 6-shogaol-rich extract (10 mg/kg), obtained by extracting dried ginger powder with 95% ethanol at 80 °C, showed significant attenuation of diethylnitrosamine-induced hepatotoxicity. Specifically, treatment reduced serum markers of liver injury, including aspartate aminotransferase (AST), alanine aminotransferase (ALT) and indices of lipid peroxidation [39].

Consistent findings have been reported in HepG2 cells, where the same extract (50 µg/mL) exerted a stronger antioxidant effect through activation of Nrf2 and upregulation of heme oxygenase-1 (HO-1), mediated by the p38 MAPK and PI3K/Akt signaling pathways [39]. Furthermore, in a steatotic liver model, hamsters receiving ginger ethanolic extract by gavage (800 µg 6-gingerol/kg body weight/day) exhibited reduced hepatic lipid accumulation and oxidative stress [40]. Importantly, the antioxidant capacity of ginger appears to correlate strongly with its total phenolic and flavonoid content, underscoring the central role of these bioactive compounds in mediating its hepatoprotective effects [41].

Finally, *in vitro* experimental evidence has demonstrated that ginger extracts obtained using different extraction methods exert significant antimicrobial and antioxidant activities against oral pathogens, including *Streptococcus mutans*, *Enterococcus faecalis*, *Staphylococcus* spp., and *Lactobacillus* spp., isolated from oral swabs of infected patients. These findings suggest that ginger may contribute not only to the inhibition of microbial growth but also to the mitigation of oxidative stress associated with oral infections [42].

In addition to its antioxidant effects, ginger demonstrates well-documented anti-inflammatory properties. These effects are reflected in its ability to modulate several cell signaling pathways, including NF-κB and protein kinase B (Akt), leading to the suppression of pro-inflammatory cytokines production such as TNFα and IL-6 [11,43]. Bioactive compounds of ginger, particularly gingerols and shogaols, have been shown to inhibit major inflammatory mediators, including cyclooxygenase-2 (COX-2) and inducible nitric oxide synthase (iNOS), while also reducing leukocyte infiltration. Collectively, these mechanisms contribute to decreased tissue swelling and inflammation, thereby supporting the alleviation of the inflammatory conditions [44].

Accordingly, short-term ginger supplementation is increasingly recognized as a potential supportive approach in the management of chronic inflammatory conditions. Building on its well-established cellular and molecular anti-inflammatory effects, evidence from both preclinical and clinical studies indicates that ginger may help alleviate inflammatory disorders, with particularly promising outcomes reported in rheumatoid arthritis (RA) [45]. Interestingly, oral gavage with ginger extract (50 mg/kg/day) in female rats with experimental induced rheumatoid arthritis model demonstrated anti-arthritic action effects associated with the modulation of NF-κB activity and the Wnt signaling pathway [45]. Similarly, a clinical study in patients with active RA reported that supplementation with 1500 mg of ginger per day (2 capsules, each containing 750 mg of ginger powder) reduced inflammatory markers, including serum C-reactive protein (CRP) and IL-1β gene expression. These findings suggest that ginger supplementation may help attenuate the inflammatory response observed in patients with RA [46].

Moreover, through the modulation of immune cell signaling and functional responses, ginger has emerged as a potential immunomodulatory agent. Experimental evidence indicates that its bioactive constituents can regulate the activity of multiple immune cell populations, including macrophages, neutrophils, dendritic cells, and T lymphocytes, thereby influencing both innate and adaptive immune pathways. These immunomodulatory effects have been associated with enhanced host defense mechanisms, reduced susceptibility to infection, attenuation of immune-mediated tissue damage, and potential adjunctive benefits in the management of chronic inflammatory disorders [11].

## 5. Anti-Apoptotic Actions of Ginger

The anti-apoptotic actions of ginger are particularly pronounced under toxic or oxidative stress conditions, where its bioactive compounds can reduce the process of programmed cell death (apoptosis). In several experimental models of liver and kidney disease, ginger extracts have demonstrated significant cytoprotective effects [47,48].

Moreover, zingerone has been reported to inhibit arsenic-induced hepatic tissue damage and apoptosis, *in vivo*. In male mice treated with zingerone (25, 50, and 100 mg/kg, oral gavage for 29 days) prior to arsenic administration (10 mg/kg, oral gavage for 29 days), a marked cytoprotective effect against arsenic-mediated hepatotoxicity was observed. This protection was associated with the prevention of increased apoptosis-related markers, including caspase-3 protein expression [47].

Similarly, the cytoprotective effect of ginger extract was also reported *in vivo* against cadmium-induced renal toxicity. In rats, ginger treatment at doses of 100 and 200 mg/kg significantly attenuated cadmium-mediated nephrotoxicity by protecting renal cells and preventing renal tissue and DNA damage associated with apoptosis [48].

Furthermore, the effect of a 6-gingerol-rich extract on oxidative stress, inflammation, and apoptosis modulation has been investigated in a lead acetate-induced neurotoxicity animal model. In this study, male rats administered the extract at doses of 100 and 200 mg/kg following exposure to lead acetate (7.5 mg/kg) showed marked attenuation of neurotoxicity and brain tissue damage. These protective effects were associated with increased cell survival and tissue repair, mediated through stabilization of mitochondrial function and suppression of apoptotic signaling pathways [49].

Consistent with these findings, ginger extracts have also demonstrated anti-apoptotic and hepatoprotective effects *in vitro*. In HepG2 cells exposed to acetaminophen-induced hepatotoxicity (40 mM), treatment with red ginger extract (5, 25, 125 µg/mL) decreased the levels of pro-inflammatory cytokines IL-1β and IL-6, while increasing the anti-inflammatory cytokine IL-10 compared with the positive control. Moreover, the extract downregulated the expression of apoptosis-related genes, including caspase-3, caspase-9, and JNK [50].

At the renal level, ginger bioactive compounds have also been associated with reduced apoptosis and necrosis, accompanied by beneficial modulation of both antioxidant and inflammatory pathways, ultimately leading to improved renal function markers. Accordingly, growing preclinical and clinical evidence support the potential of ginger to preserve renal function and improve fibrosis or functional impairments [51,52,53].

In particular, for an *in vivo* model of vancomycin-induced nephrotoxicity, intraperitoneal administration of vancomycin (200 mg/kg body weight for one week) in rats increased inflammatory markers such as NF-kB, IL-1β, and TNF-α, while activating the apoptotic pathway through elevated expression of Bax and caspase-3. Treatment with zingerone (25 and 50 mg/kg of body weight) exerted a cytoprotective effect, attenuating nephrotoxicity and modulating both inflammatory and apoptotic responses [52].

Clinical evidence further indicates potential metabolic benefits of ginger in renal disease. In a randomized, double-blind, placebo-controlled trial involving patients with chronic renal failure undergoing peritoneal dialysis, daily supplementation with 1000 mg of ginger was found to significantly reduce serum fasting glucose levels, a critical risk factor for cardiovascular disease and mortality in this population [53].

Additionally, ginger’s anti-apoptotic activity extends to experimental models of diabetes and oxidative damage. In a rat model of streptozotocin-induced type 1 diabetes (single intraperitoneal injection, 40 mg/kg), oral administration of ginger extract (200 mg/kg daily for 30 days) significantly reduced hepatic apoptotic markers, including Bax and p53, as demonstrated by both histological assessment and mRNA expression analysis. Concurrently, an up-regulation of the anti-apoptotic protein Bcl-2 was observed. These findings suggest that ginger extracts mitigate diabetes-associated hepatic injury through the modulation of inflammatory and apoptotic signaling pathways, thereby reinforcing its hepatoprotective role [54].

Further evidence of ginger’s cytoprotective properties through apoptosis modulation is provided by studies on 6-shogaol, a ginger-derived bioactive compound. This compound has been shown to protect human dermal fibroblasts against UVA-induced oxidative stress, enhancing cell viability, inhibiting pro-apoptotic proteins such as Bax, caspase-3, and caspase-9, and promoting antioxidant protein expression [55].

Ginger also appears to exert a cytoprotective effect at the endothelial level. In an *in vitro* model of cardiovascular diseases, 6-shogaol was shown to protect endothelial cells from oxidized LDL-induced apoptosis by reducing ROS production and inhibiting caspase-9 and caspase-3 activation, and upregulating the antiapoptotic protein Bcl-2. These findings were demonstrated in human umbilical vein endothelial cells (HUVECs) treated with oxidized LDL (200 μg/mL) and pretreated with 6-shogaol (1 μM, 5 μM, 10 μM, and 30 μM), suggesting a protective role against endothelial apoptotic injury [56].

Finally, ginger’s anti-apoptotic activity has also been documented in animal models of neurodegenerative diseases. In an epileptic mouse model, zingerone administration reduced hippocampal apoptosis, as evidenced by decreased caspase-3 expression and increased Bcl-2 levels, ultimately promoting neuronal survival. These findings indicate that zingerone may exert neuroprotective effects by reducing oxidative stress and inflammation and by inhibiting mitochondrial-mediated apoptotic pathways [57].

### Context-Dependent Apoptotic Effects of Ginger

It is important to note that the modulatory effects of ginger on apoptosis are context-dependent. While ginger exhibits anti-apoptotic properties in normal, non-cancerous tissues, certain bioactive compounds derived from ginger have been shown to selectively induce apoptosis in cancer cells, including breast carcinoma cells [58].

In particular, the treatment of the breast cancer cell lines MDA-MB-231 and MCF-7 with 6-gingerol resulted in increased production of both cellular and mitochondrial reactive oxygen species (ROS), thereby enhancing the DNA damage response through activation of the ataxia–telangiectasia mutated (ATM) kinase and p53 signaling pathways. In these cells, 6-gingerol has also induced G0/G1 cell cycle arrest and promoted mitochondrial-mediated apoptosis by modulating the Bax/Bcl-2 ratio and stimulating cytochrome c release [58].

A growing body of evidence further highlights the pro-apoptotic potential of ginger, particularly its active compound 6-shogaol, against a variety of cancer types, including ovarian and colorectal cancers [59].

Overall, the regulatory influence of ginger on apoptotic pathways appears to be highly cell-type specific and context-dependent. In healthy cells, the antioxidant properties of ginger help strengthen mitochondrial integrity, thereby preventing premature cell death. Accordingly, ginger extracts and their derivatives consistently demonstrate cytoprotective effects under physiological conditions, primarily through the reduction in oxidative stress and the suppression of pro-apoptotic markers such as caspase-3 and Bax.

Conversely, in the metabolically stressed environment typical of tumor cells, these same compounds may induce a pro-oxidant shift or interfere with key survival signaling pathways, ultimately promoting apoptotic cell death. Consequently, ginger should be regarded not as a universal inhibitor of apoptosis, but rather as a selective and context-dependent modulator of programmed cell death.

## 6. Forms of Consumption of Ginger

Ginger is consumed worldwide in a wide variety of forms and is frequently used in both traditional medicine and culinary practices, reflecting its remarkable versatility. The raw rhizome, often grated, minced, or squeezed, is commonly used to confer its intense flavor to teas, foods, and beverages. Ginger is often dried and ground into a powder, serving as a key component in various nutraceutical formulations [15,16,17,18].

It is important to emphasize that different drying processes, such as sun-drying, oven-drying, or freeze-drying, can markedly influence the concentration of bioactive compounds and, consequently, the antioxidant activity of the final product. Fresh ginger juice, obtained by extracting fresh rhizomes, has long been used in traditional medicine to treat various disorders and consumed for various health benefits [60].

Indeed, short-term consumption of ginger juice has been reported to induce significant changes in both the composition and functional activity of the gut microbiota [61,62]. In addition to the traditional preparations, standardized ginger extracts are commercially available as dietary supplements and are frequently used in clinical settings, particularly for the management of various gastrointestinal conditions, such as nausea and dyspepsia [61]. For therapeutic applications, ginger extracts, oleoresins, and essential oils are often encapsulated to ensure standardized dosing and enhanced stability of their bioactive ingredients [63].

This wide range of consumption patterns, from simple food ingredients to concentrated extracts, supports the use of ginger in a wide range of health conditions, including nausea and vomiting, gastrointestinal dysfunction, pain, inflammation, and metabolic syndrome, among others [22].

Although ginger is generally regarded as safe, the use of standardized extracts, whether administered alone or in combination with other therapies, requires careful consideration of potential adverse effects and drug interactions. Furthermore, as discussed above, both the drying technique and the extraction solvent can significantly influence the final concentration and phytochemical profile of bioactive compounds, thereby modulating the biological activity and the clinical effectiveness of the final product [60,64]. Collectively, these factors highlight the critical need for optimization and standardization of preparation protocols to ensure reproducible quality and consistent therapeutic outcomes.

## 7. Emerging Research: Nano-Formulations, Bioavailability Enhancement of Ginger

Despite these promising pharmacological properties, the therapeutic use of ginger faces significant challenges. Primarily, the poor bioavailability and chemical instability of its active compounds. The hydrophobic nature of gingerols and related constituents, coupled with their susceptibility to degradation, limits systemic absorption and leads to rapid metabolic breakdown in the gastrointestinal tract. Furthermore, the resulting variability in pharmacokinetic profiles produces unpredictable plasma concentrations, complicating the translation of preclinical efficacy into effective clinical therapies.

These limitations highlight the pressing need for innovative delivery systems to overcome the challenges associated with solubility, stability, and targeted tissue distribution of ginger compounds to optimize both stability and delivery efficiency. In this context, nanotechnology provides a promising mechanistic strategy to overcome the inherent lipophilicity and rapid metabolism of gingerols, offering novel strategies to exceed the intrinsic constraints of phytochemicals like those found in ginger. Newer nanoformulations use carriers such as liposomes, polymeric nanoparticles, phytosomes, and nanoemulsions to enhance solubility, chemical stability, and cellular penetration of bioactive compounds. Such approaches expand the traditional applications of phytochemicals and increase their therapeutic potential by improving their pharmacokinetic and pharmacodynamic profiles [65].

Different nanoformulation approaches have been developed to optimize the efficacy of ginger bioactive compounds. Among these strategies, chitosan-based nanoparticles produced through ionic gelation have attracted considerable attention. In this context, nanoparticles loaded with *Zingiber officinale* essential oil have been successfully developed.

These nanoparticles displayed spherical shape, with an encapsulation efficiency ranging from 49.11% to 68.32%, a loading capacity of 21.16–27.54%, and particle sizes between 198.13 and 318.26 nm. Their antimicrobial activity was evaluated *in vitro* using an agar well diffusion assay against selected bacterial strains. Importantly, these nanoparticles were positively charged, so they can easily interact with the negatively charged mucus layer and thereby improve the bioavailability of bioactive compounds [66].

Similarly, Ahmad et al. developed a 6-Gingerol-nanoemulsion coated with chitosan using an aqueous titration method. This formulation was subsequently characterized in terms of morphology, thermodynamic stability, *in vitro* release profile (*in vitro*), mucoadhesive properties, and ex vivo nasal permeation. The results suggested that such nano-formulations may enhance brain bioavailability following intranasal administration, owing to their mucoadhesive properties and their ability to facilitate transport across key physiological barriers, including the intestinal barrier and the blood–brain barrier (BBB) [66,67].

Moreover, ginger extracts have also been incorporated into phytosomal formulations, alone or in combination with other plant-derived bioactive compounds, such as those obtained from *Rosa canina* L. These formulations act synergistically to reduce inflammation, while the phospholipid complexes characteristic of phytosomes improve the bioavailability and absorption of the encapsulated phytochemicals, providing a stable formulation that ensures an improved bioavailability and biodistribution of the active compounds and consequently increases their antioxidant and anti-inflammatory action [68].

In this context, the study by Deleanu et al. demonstrated that phytosomal formulations combining ginger extract with rosehips extract produced a 2.6-fold increase in the plasma levels of 6-gingerol and over 40% increase in the liver and kidneys concentrations compared with non-formulated extracts. Conversely, a 65% reduction in 6-gingerol accumulation was observed in the stomach. These effects were reported in C57Bl/6J mice, in which the formulations were administered by gavage prior to lipopolysaccharide (LPS)-induced systemic inflammation, highlighting the potential of phytosomal delivery to enhance tissue bioavailability and therapeutic efficacy [68].

Collectively, these advanced delivery systems significantly improve the pharmacokinetic properties of ginger bioactive compounds, resulting in enhanced efficacy and potentially allowing lower therapeutic doses compared with traditional preparations. Such approaches therefore represent a substantial improvement in delivery efficiency relative to conventional ginger preparations. Altogether, the above-illustrated delivery methods illustrate how nanotechnology can modulate the pharmacological profile of ginger to meet a variety of therapeutic targets. Essential insights into the characterization, stability, and biological effectiveness of these nanoformulations have been gained from extensive *in vitro* and *in vivo* studies [68,69,70,71].

Animal models of neurological, inflammatory, and oncological diseases have also been extensively used to investigate the biodistribution and pharmacokinetics of ginger nanoformulations. These studies have evaluated tissue accumulation and plasma concentrations, showing that nanoformulated ginger can produce beneficial effects such as tumor regression and improved neurobehavioral outcomes. Interestingly, delivery via nanocarrier systems compared to conventional extracts enhances systemic exposure and enables more precise tissue-targeted distribution, thereby strengthening the translational potential of these advanced nanoformulations [65,71].

In particular, the *in vitro* anti-proliferative and anti-inflammatory properties of encapsulated 6-gingerol have been assessed in healthy human peritoneal ligament (PDL) fibroblasts and MDA-MB-231 breast cancer cells. In this formulation, the encapsulation efficiency and loading capacity of 6-gingerol reached 25.23% and 2.5%, respectively.

Nanoliposomal encapsulation has significantly improved formulation stability, with the nanoliposomes remaining stable for up to 30 days at 4 °C, while preserving the biological activity of the encapsulated compound. This was demonstrated by its sustained anti-proliferative effects on cancer cell lines and the inhibition of IL-8 production, indicating that nanoliposomal delivery enhances both the stability and functional efficacy of 6-gingerol [71].

Preclinical studies in animal models further indicate that ginger nanoformulations are well tolerated and may exhibit lower toxicity compared with conventional preparations. A study conducted in different mouse models of colitis characterized a specific population of nanoparticles derived from edible ginger and demonstrated their efficient colon targeting following oral administration. These nanoparticles, characterized by a high concentration of ginger’s principal bioactive compounds, 6-gingerol and 6-shogaol, were found to be highly biocompatible and nontoxic, with cellular uptake occurring predominantly in intestinal epithelial cells and macrophages [72]. In addition, the improved physicochemical stability of these new standardized preparations may offer sustained efficacy under physiological conditions, while reducing the risk of generating potentially harmful degradation products during treatment [73].

Ginger-based nanoformulations have recently been proposed as promising therapeutic strategies for a wide range of pathological conditions, including infectious diseases. In this context, ginger-derived nanoparticles demonstrate promising antibacterial activity and represent a potential anti-infective agent. Furthermore, comparative analyses with other natural compounds indicate that ginger nanoformulations display excellent biocompatibility and exhibit multi-target therapeutic efficacy [74,75]. Together, these findings underscore the necessity for comprehensive toxicity and safety assessments to validate the suitability of ginger nanoformulations for human use and to facilitate their regulatory approval, critical steps toward enabling future clinical evaluation.

Table 1 provides a summary of ginger-based nanoformulations developed to enhance bioavailability.

### Limitations and Translational

Despite the enhanced efficacy of ginger-based nanoformulations, the translation from laboratory research to clinical application faces significant scale-up challenges, particularly in maintaining batch-to-batch consistency in particle size and encapsulation efficiency. In addition, stability during long-term storage—especially for lipid-based delivery systems—continues to represent a critical limitation. From a regulatory perspective, the safety profile of synthetic nanocarriers and their potential long-term toxicity require more rigorous evaluation and standardized evaluation prior to clinical implementation.

To fully exploit the therapeutic potential of ginger and related plants, and to expand the field of phytomedicine, future research must rely on a comprehensive multidisciplinary framework supported by robust pharmacological and clinical investigations.

This integrative approach is essential for optimizing the bioavailability and achieving site-specific delivery of natural bioactive compounds. Therefore, the convergence of pharmacology, phytotherapy, and nanotechnology is crucial to redefining the therapeutic potential of natural products.

## 8. Potential Health Benefits of Ginger

### 8.1. Cardiovascular Health Benefits of Ginger

Cardiovascular diseases (CVD) represent one of the most prevalent and debilitating groups of disorders worldwide, encompassing conditions that affect the heart and vasculature, including myocardial infarction, stroke, and heart failure. The pathogenesis of CVD is closely associated with atherosclerosis and is influenced by a wide range of modifiable risk factors, such as smoking, hypertension, and diabetes mellitus, and non-modifiable risk factors, including age, sex, and genetic predisposition [76].

Given the adverse effects associated with many synthetic pharmaceuticals and the suboptimal efficacy of current treatments for CVD, research has increasingly focused on identifying natural compounds capable of alleviating symptoms and modulating the underlying pathophysiological mechanisms. In this context, bioactive phytochemicals found in ginger have attracted growing interest due to their potential cardioprotective properties. Accumulating evidence indicates that ginger may contribute reduce blood pressure, lower the risk of coronary heart disease, and exert antiplatelet effects, thereby potentially inhibiting platelet aggregation and thrombus formation [17].

Several studies have reported that ginger consumption produces significant reductions in systolic and/or diastolic blood pressure [77,78]. However, other investigations have failed to demonstrate comparable antihypertensive effects [79,80,81]. The inconsistency in these findings may largely reflect substantial heterogeneity between studies, including differences in participant characteristics, including baseline blood pressure, age, sex distribution, and the presence of metabolic comorbidities, which may influence responsiveness to ginger supplementation [81,82,83].

Additionally, variability in dosage, duration of intervention, and formulation (e.g., powdered rhizome, crude extract, or standardized preparations containing differing concentrations of bioactive compounds such as gingerols and shogaols) may affect therapeutic outcomes [81,82]. Methodological differences, including sample size, study design, and blood pressure assessment methods, may also contribute to divergent results [81,83]. Notably, meta-analytic evidence indicates that ginger may exert more pronounced anti-hypertensive effects in individuals with elevated baseline blood pressure or when administered at higher doses and for extended periods [82].

Therefore, these observations suggest that while ginger appears to be a promising complementary approach for cardiovascular health, further well-designed, large-scale clinical trials are needed to clarify its efficacy and to establish optimal dosing and administration parameters.

#### Mechanisms, Preclinical and Clinical Evidence

Additional experimental research studies support ginger’s cardiovascular effects. The intravenous administration of crude ginger extract in rats induced a dose-dependent (0.3–3 mg/kg) reduction in blood pressure [84]. In this regard, the extract seems to act through a dual inhibitory effect mediated via stimulation of muscarinic receptors and blockade of Ca^2+^ channels, thus providing a mechanistic explanation for the use of ginger in hypertension [85].

Another study investigating the effect of ginger consumption in a hypercholesterolemic rat model has reported that diets supplemented with *Zingiber officinale* (2% and 4%) for 3 days have significantly increased plasma high-density lipoprotein-cholesterol levels compared with the control group. This suggests that ginger was able to modulate the renin–angiotensin system through the regulation of angiotensin-converting enzyme activity, thus influencing the vascular constriction [86].

Although the molecular mechanisms underlying the anti-hypertensive effects of ginger remain incompletely understood, its antioxidant properties appear to play a significant role [87]. In an *in vivo* study, treatment of alcohol-fed rats with 6-gingerol (10 mg/kg body weight) effectively protected alcohol-induced ROS-mediated cardiac tissue damage, an effect that is likely due to its potent antioxidant activity [88]. Furthermore, in a murine model of diabetic cardiomyopathy, the administration of 6-gingerol significantly attenuated cardiac injury by suppressing inflammatory and oxidative stress pathways. This cardioprotective effect was mechanistically associated with the activation of the Nrf2 signaling pathway [89].

Lipid peroxidation is known to induce vasoconstriction and contribute to increased blood pressure [90]. Several studies have reported that ginger, which is rich in phenolic compounds such as gingerols, shogaols, zingerone, and paradol, possess potent antioxidant properties and can decrease blood pressure by reducing lipid peroxidation [81,91].

In addition, the phenolic compounds of ginger exert vasodilatory effects, partially by increasing the plasma NO concentration [92,93]. An *in vivo* study by Wu et al. demonstrated that ginger crude extract (GCE) exerts strong vasoprotective effects and free radical-scavenging activities in porcine coronary arteries. Specifically, GCE induced endothelium-dependent relaxation of coronary vessels and enhanced vasoprotection through mechanisms involving NO synthase activation and cyclooxygenase inhibition [93]. The cardioprotective potential of ginger has also been investigated in the context of diabetes-induced cardiomyopathy. In a streptozotocin-induced diabetic rat model, treatment with ginger extract significantly reduced myocardial fibrosis and inflammation [94].

These beneficial effects were associated with decreased expression of the angiotensin II type 1 (AT1) receptor, transforming growth factor (TGF)-β1, and TGF-β3. Angiotensin II, a well-established profibrotic and proinflammatory mediator, activates the TGF-β signaling pathway, thereby promoting myocyte necrosis, hypertrophy, and fibrosis. Given the central role of the renin–angiotensin system and the SMAD/TGF-β pathway in the development of cardiomyopathic fibrosis and inflammation, the findings are particularly promising. They highlight the potential of ginger extract to attenuate diabetes-associated cardiac inflammation and fibrosis, and to contribute to the prevention of related complications [95].

The injection of labetalol-treated female albino rats with the aqueous ginger extract (200 mg/kg) resulted in marked improvements in fetal cardiac tissue integrity, along with reductions in DNA damage and apoptosis rates. This indicates that ginger extract may represent a potential therapeutic agent for attenuating labetalol-induced cardiotoxicity in the fetal heart, confirming its cardioprotective effects against myocardial damage [96]. In this context, ginger has been shown to exert cardioprotective effects in rat models of myocardial damage induced by isoproterenol and cisplatin [97,98]. Subbaiah et al. further confirmed that ginger exerts a protective effect against alcohol-induced myocardial damage by suppressing hyperlipidemia and reducing cardiac biomarkers associated with myocardial injury [99]. Additionally, 6-gingerol improves cardiac function and attenuates pressure overload-induced cardiac remodeling in C57BL/6 mice subjected to transverse aortic constriction, acting through a p38-dependent pathway [100].

Another active component of ginger, [8]-gingerol, has demonstrated anti-myocardial ischemic effects, likely mediated through multiple mechanisms, including the reduction in oxidative stress, inhibition of cardiomyocyte apoptosis via the modulation of the MAPK signaling pathway, and regulation of Ca^2+^ homeostasis by the modulation of L-type Ca^2+^ channels current. In this regard, *in vivo* experimental model of isoproterenol-induced myocardial ischemia in rats, intraperitoneal administration of 8-gingerol (10 and 20 mg/kg/day) prevented cardiomyocyte apoptosis by inhibiting the MAPK signaling pathway [101].

It is well documented that the activation of c-Jun N-terminal kinase (JNK) and nuclear translocation of NF-κB are implicated in ischemia/reperfusion injury (I/R), and elevated levels of HMGB1 and HMGB2 have been observed in the serum and myocardial tissue of patients with myocardial infarction [102]. Consistently, findings by Zhang et al. indicated that the treatment of human AC16 cardiomyocytes with 6-gingerol (10–20 μM) was able to protect I/R-induced cardiomyocyte apoptosis by modulating the JNK/NF-κB pathway and regulating HMGB2 expression [103].

Taken together, these studies not only elucidated key mechanisms underlying the therapeutic effects of ginger and its active constituents but also highlighted their potential effectiveness as promising therapeutic agents for the eventual future clinical management of cardiovascular diseases.

### 8.2. Neuroprotective Effects of Ginger

The ageing population faces an escalating burden of neurodegenerative disorders, with Alzheimer’s disease (AD) alone affecting more than 26 million individuals worldwide, a number projected to quadruple by 2050 [104]. At present, no effective treatments exist for most age-related neurodegenerative diseases. Consequently, prioritizing the preventive strategies and identifying new nutraceuticals and therapeutic agents has become essential. In this context, considerable research has focused on plant-derived bioactive compounds, many of which possess well-documented protective properties, including anti-inflammatory and antioxidant effects relevant to neurodegenerative diseases, particularly by targeting neuroinflammation. These benefits are particularly linked to their ability to target neuroinflammation, a central pathogenic mechanism that drives the development and progression of diseases such as Parkinson’s disease (PD), AD, and multiple sclerosis (MS) [10]. Chronic neuroinflammation is a central pathogenic mechanism shared by neurodegenerative diseases. It is predominantly mediated by activated microglia, which release pro-inflammatory mediators including TNF-α, IL-1β, NO, and ROS. Persistent microglial activation promotes neuronal apoptosis and accelerates disease progression [11]. Plant-derived bioactive compounds have garnered considerable research interest due to their potential to target neuroinflammation. Among these, ginger and its principal bioactive constituents have demonstrated multi-target neuroprotective activity. Ginger is classified as Generally Recognized as Safe (GRAS) by the U.S. FDA, supporting its potential as a nutraceutical in the context of neurodegenerative disorders [105,106]. The investigations of the mechanisms of action of ginger-derived bioactive compounds, such as 6-gingerol, 10-gingerol, and 6-shogaol, have identified multiple neuroprotective pathways [11].

Ginger-derived bioactive compounds exert neuroprotective effects by modulating multiple disease-relevant targets simultaneously [107]. In a study by Simon et al. (2020) [108], a parallel artificial membrane permeability assay for the BBB, previously validated for natural compounds, was used to investigate whether gingerol and shogaol derivatives could passively diffuse across the BBB. The results showed that [6]-gingerol, [8]-gingerol, and [6]-shogaol were able to cross the BBB via passive diffusion, suggesting that they contribute to the positive effects of ginger extracts in the CNS [108].

The principal mechanisms identified to date include potent anti-inflammatory activity characterized by the inhibition of NF-κB activation via blockade of IκB phosphorylation, resulting in reduced transcription of pro-inflammatory mediators such as TNF-α, IL-1β, IL-6, and COX-2 [109,110]. The antioxidant activity also contributes to these neuroprotective effects through upregulation of the Nrf2 transcription factor and restoration of endogenous antioxidant enzymes, including superoxide dismutase (SOD), catalase (CAT), glutathione S-transferase (GST), and glutathione peroxidase [111,112,113,114,115]. In addition, ginger-derived compounds modulate apoptotic pathways by regulating the Bax/Bcl-2 ratio, mitochondrial membrane potential, caspase-3 activity, and inhibiting the activation of NLRP3 inflammasome [107,116].

Finally, regulation of microglial activity represents a critical mechanism underlying the neuroprotective effects of ginger-derived compounds. These effects include the suppression of microglial hyperactivation and the consequent attenuation of neuroinflammatory signaling, mediated in part through inhibition of the SAPK/JNK pro-apoptotic pathway and upregulation of survival pathways, such as survivin expression [117,118].

In addition, ginger-derived exosomes (G-EX) have emerged as promising naturally derived nanovesicles for drug delivery. These vesicles have been shown to improve drug penetration, including improved transcorneal permeation. Furthermore, ginger-based nanovesicles enable controlled and sustained drug release for up to 48 h, which consequently enhances drug bioavailability and improves overall therapeutic efficacy [115].

Despite their distinct etiologies, AD, PD, and MS share convergent pathogenic mechanisms characterized by neuroinflammation and oxidative stress, which are consistently targeted by ginger-derived bioactive compounds. Across these disorders, the NF-κB/IκB axis and Nrf2-mediated antioxidant pathway emerge as key molecular targets, while modulation of microglial cell activation represents the common cellular effector mechanism. This mechanistic convergence suggests that ginger-derived compounds may exert broad-spectrum neuroprotective effects rather than acting in a disease-specific manner [119].

Furthermore, the gut–brain axis provides an additional therapeutic dimension. This interaction is particularly relevant in PD, where intestinal dysbiosis often precedes central neurodegeneration, but it is increasingly recognized as a contributing factor in the pathophysiology of AD and MS.

In this context, ginger-derived exosome-based delivery systems represent an innovative translational strategy that integrates the intrinsic anti-inflammatory properties of natural compounds with improved bioavailability and targeted delivery following oral administration [120].

#### 8.2.1. Ginger in Alzheimer’s Disease

AD is a slowly progressive neurodegenerative disorder characterized by the accumulation of extracellular amyloid plaques and intracellular neurofibrillary tangles. These pathological hallmarks arise from the accumulation of Aβ in the medial temporal lobe and neocortical structures, leading to synaptic dysfunction and neuronal loss. Current research focuses on understanding the molecular mechanisms underlying AD pathology [121].

Ginger and its bioactive constituents are being investigated as potential alternative therapeutic agents capable of slowing or modifying disease progression. Several *in vitro* studies have demonstrated that pretreatment with 6-gingerol reduces Aβ-induced oxidative and nitrosative stress in neuroblastoma cells. These effects are mediated through the modulation of mitochondrial membrane potential, regulation of the Bax/Bcl-2 ratio, attenuation of DNA fragmentation, and suppression of caspase-3 activity. In PC-12 cells, 6-gingerol also inhibited Aβ1-42-induced apoptosis via downregulation of GSK-3β and upregulation of Akt, thereby initiating a neuroprotective signaling cascade [122]. In addition, ginger extracts have been reported to directly inhibit Aβ aggregation and protect neuronal cells from Hydrogen peroxide (H_2_O_2_)-induced oxidative damage *in vitro* [114,115].

More recently, a study by Pan et al. (2024) [123] identified 6-gingerol as the most pharmacologically active constituent of ginger for AD. The study demonstrated that 6-gingerol exhibits the highest binding affinity toward key molecular targets, including Acetylcholinesterase, Matrix metalloproteinase-2 (MMP2), and COX-2, with Caspase-3 (CASP3) identified as a central hub gene in the associated interaction network. Molecular dynamics simulations further confirmed stable ligand–target binding interactions, supporting a potential regulatory role of 6-gingerol in cholinesterase metabolism and apoptosis-related signaling pathways [123].

Preclinical studies in rodent models have demonstrated that ginger administration significantly suppressed the mRNA expression of pro-inflammatory cytokines and endothelial adhesion molecules, including TNF-α, IL-1β, COX-2, MIP-1α, and MCP-1 [120]. Additionally, increased levels of nerve growth factor in the hippocampus have triggered the activation of ERK and CREB signaling pathways, thereby promoting synaptogenesis and neurite outgrowth [118,121]. In an AD mouse model, Zahedi et al. reported that oral administration of ginger extract reduced TNF-α and IL-1β concentrations in both blood and brain, attenuated Aβ deposition in hippocampal tissue, enhanced gut microbiota activity, and reinforced the intestinal barrier integrity via upregulation of tight junction proteins. Collectively, these effects were associated with an improved performance in learning and memory tasks compared with the untreated group [124].

Watari et al. conducted an open-label, crossover clinical trial to investigate the effects of *kihito*, a traditional Japanese Kampo formula comprising 12 herbs, including *Zingiber officinale*, on cognitive function in patients with AD. Ten participants completed the 32-week study, receiving either *kihito* extract combined with acetylcholinesterase inhibitors, or acetylcholinesterase inhibitors alone during alternating 16-week periods. The study showed a trend toward reduced cognitive decline, although results did not reach statistical significance. The authors proposed potential mechanisms, including inhibition of calcium influx and tau dephosphorylation. However, the specific contribution of ginger within the multi-herb formula could not be isolated [125]. In another clinical investigation, Saenghong et al. conducted a double-blind trial enrolling 60 healthy middle-aged Thai women, who were randomly assigned to receive a placebo or standardized ginger extract at doses of 400 mg or 800 mg daily for two months.

Cognitive performance was assessed using electrophysiological measures with a computerized working memory battery. Participants receiving 800 mg of ginger extract exhibited significant improvement in working memory performance across multiple cognitive domains, while dose-dependent improvements were also observed in those receiving 400 mg. These findings suggest that ginger may modulate monoaminergic and cholinergic neurotransmitter systems, potentially through inhibition of cholinesterase by its principal bioactive compounds, 6-gingerol and 6-shogaol, in addition to its antioxidant properties [126]. Together, these studies indicate promising cognitive-enhancing properties of ginger-containing preparations. Nevertheless, larger, longer-duration, and more rigorously controlled trials are required to confirm therapeutic efficacy and to further elucidate the underlying mechanisms of action.

#### 8.2.2. Ginger in Parkinson’s Disease

PD is currently recognized as the fastest-growing neurodegenerative disorder worldwide. In most cases, disease progression is associated with the misfolding and aggregation of α-synuclein into oligomers, fibrils, and Lewy bodies. However, the initial triggers of this aggregation process remain poorly understood. In certain individuals, genetic mutations contribute to the disease onset, while in others, additional pathological mechanisms may drive neurodegeneration with or without direct involvement of α-synuclein [127].

*In vitro* studies provide evidence for the neuroprotective potential of ginger-derived compounds. In PC12 cells exposed to the dopaminergic neurotoxin 6-hydroxydopamine (6-OHDA), 6-gingerol exerted a significant neuroprotective activity by inhibiting pro-apoptotic SAPK/JNK signaling pathway, while simultaneously activating pro-survival mechanisms through upregulation of survivin, an inhibitor of apoptosis protein family member. These results demonstrate a direct anti-apoptotic mechanism in dopaminergic neurons [117]. Evidence from animal models further supports these effects. In a mouse model of PD induced by MPTP, treatment with 6-shogaol significantly improved both motor deficits and depressive-like behaviors. Neurochemical analysis revealed restoration of key neurotransmitters, including dopamine, serotonin, norepinephrine, and GABA levels in the striatum and hippocampus, normalizing the monoamine neurotransmitter imbalance characteristic of PD [127].

A complementary study by Kim et al. investigated the co-administration of 6-shogaol with levodopa, addressing a major limitation of levodopa monotherapy, its inability to modify neuroinflammation-driven disease progression. The combined treatment produced synergistic effects: levodopa improved motor symptoms while 6-shogaol suppressed microglial activation and reduced expression of TNF-α, IL-1β, IL-6, and ROS, resulting in attenuated dopaminergic neuronal damage, an effect not achieved by either compound alone [118].

Within the context of the gut–brain axis, 6-shogaol has also been shown to suppress intestinal barrier dysfunction and inhibit α-synuclein aggregation in both intestinal and brain tissues, while reducing TNF-α-mediated pro-inflammatory signaling [120,128]. Furthermore, Cui et al. have developed Exo@tac, acid-resistant ginger-derived exosome-like nanovesicles loaded with tetrahedral framework nucleic acids (TFNAs) designed for oral administration. These nanovesicles were able to normalize gut microbiota composition and regulate the microbiome-gut–brain axis (MGBA), providing a novel biodegradable delivery platform with potential therapeutic applications in PD management [126,129].

#### 8.2.3. Ginger in Multiple Sclerosis

Multiple Sclerosis (MS) is a chronic, immune-mediated disorder of the CNS. MS is characterized by demyelination, axonal degeneration, and persistent neuroinflammation, which progressively lead to both physical and cognitive impairments. Although substantial progress has been made in developing disease-modifying therapies aimed primarily at suppressing inflammatory activity, no curative treatment is currently available [130].

An *in vitro* study by Han et al. demonstrated that 6-gingerol suppressed LPS-induced dendritic cell (DC) activation and induced immunological tolerance *in vitro*. This targeted immunomodulatory effect specifically reduced the capacity of DCs to activate autoreactive T cells, providing a mechanistic basis for reduced CNS inflammation [131,132].

Furthermore, ginger extract enhanced the activity of Regulatory T cells (Tregs), promoting immune homeostasis and suppressing autoreactive T-cell responses. Additional investigations into 6-Shogaol and its metabolite 6-Paradol confirmed anti-inflammatory and immunomodulatory properties consistent with observations reported in AD and PD models [133,134].

Consistent findings have also been reported *in vivo* using the experimental autoimmune encephalomyelitis (EAE) murine model, the principal animal model for MS. In this model, ginger extract reduced the infiltration of autoreactive inflammatory cells into the CNS, attenuating neuroinflammation and demyelination [131]. Mechanistic analyses revealed modulation of a variety of pro-inflammatory cytokine gene expression, including IL-17, IFN-γ, TNF-α, IL-27, and IL-33, which play key roles in myelin destruction [133]. Furthermore, ginger extract enhanced the function of Treg cells, promoting immune homeostasis and suppressing autoreactive T-cell responses. Additional investigations into 6-Shogaoland its metabolite 6-Paradol confirmed anti-inflammatory and immunomodulatory properties consistent with observations reported in AD and PD models [133,134].

A clinical trial conducted by Foshati et al. investigated the effects of ginger supplementation in patients with relapsing-remitting MS, with a focus on gastrointestinal symptoms commonly associated with the disease. Ginger’s supplementation produced clinically relevant antiemetic and prokinetic effects, reducing the frequency and severity of constipation, nausea, and abdominal pain. These effects are mediated peripherally, via modulation of gastric motility by gingerols and shogaols, rather than directly targeting central immunopathological mechanisms. However, the findings support the potential use of ginger as a symptomatic adjunct to standard MS therapies [135].

**Figure 2:**
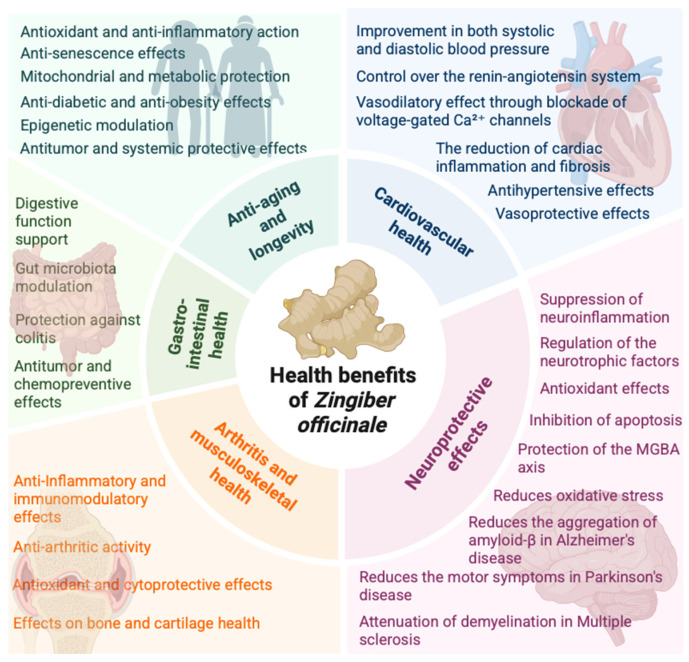
Ginger and its active compounds offer significant health benefits. At the cardiovascular level, for example, ginger helps lower blood pressure, thereby contributing to protection against heart attacks and strokes. Ginger also boasts neuroprotective effects by combatting neuroinflammation and anti-ageing effects by counteracting age-related oxidative stress. Ginger has also been shown to be effective in treating arthritis and musculoskeletal pain, reducing joint pain and inflammation. Finally, ginger is a well-known gastroprotective agent that improves digestion, stimulates intestinal motility, and protects from tumors and colitis.

Despite promising findings, the substantial translational gap remains due to the limited number of human clinical trial data. Although preclinical evidence is consistent and mechanistically coherent, the transition from animal models to human disease requires adequately powered RCTs with validated biomarker endpoints. The favorable safety profile of ginger, confirmed by its GRAS status, supports the feasibility of such investigations. Future research should prioritize standardized extract formulations, dose-ranging studies, and disease-specific outcome measures to substantiate the therapeutic claims suggested by the existing literature (Figure 2).

In conclusion, ginger and its bioactive constituents represent a promising class of multi-target nutraceuticals for the prevention and management of neurodegenerative diseases. Their capacity to simultaneously modulate neuroinflammation, oxidative stress, apoptosis, and the gut–brain axis positions them as candidates for adjunctive therapeutic strategies. Rigorous clinical research is now warranted to translate the compelling preclinical evidence into evidence-based recommendations.

### 8.3. Ginger Effects in Arthritis and in Musculoskeletal Health

A considerable number of studies have comprehensively investigated the pharmacological properties of ginger and its bioactive molecules and demonstrated a significant efficacy in the management of both arthritis and the protection of overall musculoskeletal health. Therapeutic benefits have been documented for the whole ginger extracts as well as for its specific constituents, including shogaols and zingerone.

The most relevant findings include pain reduction, improved skeletal function, suppression of cytokine- and inflammation-mediated signaling pathways, protection of cartilage tissue, and enhancement of bone mineral density.

Several recently published systematic reviews have compiled evidence on the effects of ginger-derived compounds on the physiology of osteoarticular cells, as well as in *in vivo* and *in vitro* models of inflammatory diseases affecting the osteoarticular system [45,136]. Phan et al. showed that ginger extract suppressed the expression of chemokines, macrophage chemotactic factor MCP-1, and of interferon-γ-activated protein IP-10, in human synoviocytes activated with 1 ng/mL of TNFα. Both MCP-1 and IP-10 are implicated in the pathogenesis of RA and osteoarthritis. However, this regulatory effect was observed when ginger extract was used synergistically with the extract of *Alpinia galanga*, another species belonging to the Zingiberaceae family [137]. Zingerone, an important phenolic alkanone derived from dried ginger, effectively suppressed TNFα-induced proliferation, migration, ROS formation, and the transcript levels of IL-1β and IL-6 by targeting MAPKs (ERK, p38, and JNK) signaling pathways in arthritic fibroblast-like synoviocytes (FLSs). FLSs are considered key effector cells in rheumatoid arthritis [138]. Similar results were obtained using α-Cedrol, another active biomolecule compound of ginger. α-Cedrol attenuated the severity of collagen-induced arthritis (CIA) pathology in an *in vivo* DBA/1J mouse model and modulated LPS-mediated responses in FLSs *in vitro*.

Mechanistically, α-Cedrol limited ERK/MAPK and p65 NF-κB activation, reducing the mRNA levels and protein expression of COX-1, COX-2, and consequently inhibiting prostaglandin E2 (PGE2) release, and the production of pro-inflammatory mediators TNFα, and IL-1β. In addition, α-Cedrol reduced the expression of MMP-13 and MCP-1 mRNA, both implicated in osteoclastogenesis resistance [139].

Among ginger constituents, 6-shogaol is considered one of the most potent anti-inflammatory compounds and exhibits biological activity comparable to that of the whole ginger extracts. 6-Shogaol is an alkylphenol derived from ginger rhizomes, which inhibited proliferation, migration, and invasion and induced apoptosis in the human rheumatoid fibroblast-like synoviocyte line MH7A, and in RA FLSs. Moreover, it reduced the production of TNFα, IL-1β, IL-6, IL-8, MMP-2, and MMP-9. Molecular analysis indicated that 6-shogaol inhibited the PI3K/AKT/NF-κB signaling pathway through the activation of PPAR-γ. Treatment with 6-shogaol also attenuated joint destruction in mice with CIA [140].

Ginger extracts appear to have beneficial preventive effects partly through their antioxidant properties, as demonstrated *in vitro* models. Pretreatment of the C28/I2 human chondrocyte cell line with ginger extract significantly increased the gene expression of antioxidant enzymes and reduced IL-1β-induced increases in ROS, lipid peroxidation, the Bax/Bcl-2 ratio, and caspase-3 activity, without evident cytotoxicity. These findings demonstrated that the antioxidant activity of ginger extract markedly reduces IL-1β-induced oxidative stress and the subsequent mitochondrial apoptosis, which represents a major apoptotic mechanism of chondrocyte cells [141].

Furthermore, chondrocytes from normal and osteoarthritic sow cartilage explants pretreated with ginger extract under cytokine stimulation for 24 h showed a significant reduction in NO and PGE2 production [142]. Villalvilla et al. demonstrated that 6-shogaol inhibited NO production and reduced IL-6 and MCP-1 expression following TLR4 signaling induction by LPS stimulation in *in vitro* chondrocyte models. Consistent with TLR4-pathway inhibition, 6-shogaol reduced ERK1/2 phosphorylation and downregulated NOS2 and MyD88 expression. Repression of MMP2 and MMP9 activity in LPS-activated chondrogenic cell line, as well as NO production and cathepsin-K activity in human primary chondrocytes, were also observed [143].

In an *in vivo* model, a study conducted by Fouda et al. reported that a hydroalcoholic extract of *Zingiber officinale* rhizomes improved several clinical parameters, including clinical scores, disease incidence, joint temperature, swelling, and cartilage destruction in rat collagen-induced arthritis (CIA) treated daily by intraperitoneal injection. These improvements were accompanied by reductions in serum levels of IL-6, TNFα, IL-1β, IL-2, and anti-CII antibodies [144]. Similarly, the oral administration of an aqueous extract of *Zingiber officinale* root has significantly decreased the serum levels of IL-4, IFNγ, and IL-17 and inhibited the IL-17 expression in the spleen and paw tissues of mice with CIA. The extract also downregulated MMP-1, MMP-3, and MMP-13 expression in paw tissues and reduced inflammatory bone damage within the joints. In human synovial fibroblasts activated by IL-1β, the extract significantly decreased IFNγ and IL-17 production by suppressing their mRNA levels and reducing the mRNA expression of MMP-3 and MMP-13. HPLC characterization revealed that 1,4-cineol, 6-gingerol, vanillylacetone, and shogaol were the main molecules present in the aqueous ginger root extract [145]. More recently, Öz et al. demonstrated, in a rat model of CIA, that oral administration of ginger root extract (50 mg/kg/day) attenuated inflammatory and metabolic mediators involved in RA pathogenesis. Compared to untreated CIA controls, treatment decreased serum TNFα, IL-6, and IL-17 levels and reduced tissue IL-17 and NF-κB expression. The ginger-treated group also exhibited reduced serum Dickkopf-1 (DKK-1) and increased sclerostin, suggesting modulation of Wnt/β-catenin signaling [45].

In the CFA-induced arthritis rat model, dehydrozingerone (DHZ) also markedly attenuated disease severity and improved functional outcomes. Intragastric administration of 100 mg/kg of DHZ reduced the global arthritic score and lowered serum levels of alkaline phosphatase, aspartate, alanine aminotransferases, rheumatoid factor, C-reactive protein, and pro-inflammatory cytokines (TNFα, TGFβ, IL-1β, IL-6). Additionally, reductions in malondialdehyde and VEGF were observed. Antioxidant defenses were concomitantly enhanced, with elevated superoxide dismutase and glutathione levels in serum [146].

Clinical investigations have also evaluated ginger supplementation in human patients. In a randomized controlled trial, individuals with knee osteoarthritis who received 500 mg of powdered *Zingiber officinale* twice daily for 3 months showed reduced serum levels of TNFα and IL-1β [147]. A meta-analysis assessing the clinical efficacy and safety of oral ginger for symptomatic treatment of osteoarthritis (OA) reported a modest but statistically significant reduction in both pain and disability. Despite these benefits, patients receiving ginger were more likely to discontinue treatment because of adverse events, leading authors to conclude that the overall evidence quality was moderate [148].

In a more recent randomized controlled trial, the efficacy and safety of steamed ginger extract (GGE03) were evaluated in patients with mild knee osteoarthritis. Participants receiving 1600 mg/day of GGE03 showed statistically significant improvements in pain, stiffness, physical function, and patient global assessment compared with placebo. No adverse safety events were reported, suggesting that the extract may present a beneficial functional food for managing osteoarthritis symptoms [149]. Furthermore, recent randomized, placebo-controlled trials have shown that the administration of a ginger extract (125 mg/day) standardized to contain 10% total gingerols and no more than 3% total shogaols produced favorable effects on exercise-induced pain perception, functional capacity, and inflammatory markers in men and women with mild to moderate muscle and joint pain. This supplementation appeared to be well tolerated and was associated with improvement in knee range of motion, markers of health, and perceptions about quality of life in individuals with mild-to-moderate joint pain [150,151] (Figure 2, Table 2).

### 8.4. Anti-Aging and Longevity Effects of Ginger

Aging is a complex life process marked by a gradual decline in physiological functions, usually linked to a higher risk of developing disabling conditions such as neurodegenerative, inflammatory, and neoplastic diseases.

Hallmarks of aging include genomic instability, oxidative stress, epigenetic modifications, telomere shortening, loss of proteostasis, dysfunctional macroautophagy, mitochondrial dysfunction, cellular senescence, altered cell–cell communication, chronic inflammation, dysbiosis, and a reduced capacity to absorb nutrients. Among these, oxidative stress and chronic inflammation play a central role in determining genomic instability, telomere attrition, mitochondrial dysfunction, and cellular senescence.

Considering oxidative stress as a hallmark of aging, research has extensively examined the antioxidant properties of ginger and its ability to neutralize free radicals involved in oxidative processes linked to DNA damage [152]. Several *in vitro* and *in vivo* studies suggest that ginger bioactive compounds may contribute to repairing nuclear and mitochondrial DNA damage associated with genomic instability [153]. A 21-day experiment in which rats presenting bromobenzene-induced hepatotoxicity were treated with an ethanolic ginger extract demonstrated antioxidant effects by enhancing defense systems, including hepatic glutathione, SOD, GPx, glutathione reductase, and GST [154]. Similar antioxidant effects were observed in rats fed dietary ginger exposed to lindane-induced oxidative stress [155]. These results have also been corroborated by human clinical studies; Danwilai et al. found that administering 6-gingerol to cancer patients receiving chemotherapy significantly increased antioxidant enzymes when compared to a placebo [156].

Chronic inflammation, another hallmark of aging (“inflammaging”), further contributes to genomic instability and age-related tissue dysfunction [157]. In addition to its antioxidant effects, ginger also shows potential as an anti-inflammatory agent, as it inhibits lipoxygenase, COX-2, and the NF-κB pathway [30]. A study in rats demonstrated that zingerone, administered at 150 mg/kg body weight two hours before a pro-inflammatory stimulus (LPS), lowered the levels of 8-hydroxy-2′-deoxyguanosine (8-OHdG), a marker linked to inflammation and DNA damage [158]. Furthermore, ROS, an unbalanced diet, smoking, and a sedentary lifestyle contribute to the gradual shortening of telomeres, small DNA structures at the ends of chromosomes that protect the genetic material after repeated replication. Telomere attrition supports the process of cellular senescence, leading to chromosomal damage and subsequent cellular apoptosis [159,160].

The process of senescence can be a double-edged sword depending on the biological context and age. In young individuals, this process is involved in protective mechanisms such as wound healing and tissue cellular remodeling. However, in older individuals, it contributes to chronic inflammation, atherosclerosis, mitochondrial dysfunction, and neurodegenerative processes [161].

Senescence is a cellular process characterized by stable growth arrest resulting in phenotypic alterations such as metabolic reprogramming, increased autophagy, and the development of a proinflammatory secretome [162,163]. Cells involved in the senescence process produce proteases responsible for the breakdown of membrane receptors, proteins, and other structures essential for the correct functioning of the tissue environment [164]. Several age-related conditions, including sarcopenia, neurodegenerative disorders such as AD, and osteoarthritis, are regulated in part by molecular pathways associated with the activation of senescence processes [161,165].

In addition, metabolic dysfunction represents a critical contributor to the aging process. Although senescent cells are no longer able to divide, they remain metabolically active and secrete a range of bioactive molecules with pro-inflammatory and pro-tumorigenic effects. In this context, the mechanistic target of rapamycin (mTOR) pathway plays a central role in regulating cellular metabolic processes such as protein and lipid synthesis, as well as in modulating the senescence-related secretory phenotype (SASP) and autophagy [166]. Inhibition of mTOR signaling in senescent cells is a critical mechanism underlying the anti-aging interventions, as it places the cells in a quiescent cellular state, enhances autophagic processes, and limits the secretion of SASP [167].

Concomitantly, additional stress-responsive signaling pathways contribute to the initiation and maintenance of cellular senescence. In particular, the upregulation of pathways such as p38 MAPK and p16INK4a is triggered by cellular stressors, including oxidative stress, DNA damage, telomere shortening, and oncogene activation, thereby reinforcing the senescent phenotype [168].

According to the existing literature, the potent antioxidant properties of ginger could counteract the aging-related processes. Importantly, at the skeletal muscle level, 6-gingerol and 6-shogaol have been shown to activate the Nrf2 signaling pathway. Upon activation, Nrf2 dissociates from its inhibitory complex, translocates to the nucleus, and binds to antioxidant response elements (AREs), thereby promoting the transcription of genes involved in cellular antioxidant defense [169].

Notably, this treatment induced cell cycle arrest at the G0/G1 phase, with concomitant downregulation of the proliferation marker Ki67. These effects collectively suggest a reduction in replicative stress, contributing to delayed cellular senescence and enhanced muscle regenerative capacity [170].

Supporting this mechanism, a recent *in vitro* study has demonstrated that treatment of senescent primary human myoblasts in culture with ginger extracts (GE1 and GE2), containing different concentrations of 6-gingerol and 6-shogaol, showed a significant increase in cell population, accompanied by enhanced cellular differentiation and a morphological shift toward a more youthful myoblast phenotype. Interestingly, this treatment induced cell cycle arrest at the G0/G1 phase, with concomitant downregulation of the cell proliferation marker Ki67. These effects collectively suggest an arrest of myoblast division, contributing to delaying the cellular senescence and enhanced muscle regenerative capacity [170].

In line with these findings, further evidence supports the senomodulatory effects of ginger-derived compounds. Human WI-38 fibroblasts induced to senescence with ionizing radiation, treated with Gingerone A, showed a significant and selective reduction in the number of senescent cells by positive regulation of cleaved caspase 3 and negative regulation of the anti-apoptotic protein Bcl-XL [171]. A reduction in pro-inflammatory proteins (IL-6) and IP-10 (interferon-γ-induced protein 10 and an increase in anti-inflammatory proteins (IL-10 and IL-13) were also observed.

Another hallmark of senescence is mitochondrial dysfunction, which often results from abnormal formation of ROS as a result of oxidative stress. At the same time, aging organisms display reduced mitochondrial membrane potential, leading to ATP depletion, followed by further ROS and inflammatory cytokines production, leading to a progressive decline culminating in apoptosis [172]. This is associated with mitochondrial DNA (mtDNA) point mutations, which results in mitochondrial respiratory chain protein dysfunction and increased ROS production, exacerbating mitochondrial dysfunction. This phenomenon is recognized as the mitochondrial free radical theory of aging (MFRTA) [173].

A study conducted by Wang and colleagues showed that ginger enriched with gingerol, administered to diabetic rats (type 2 diabetes mellitus) for 8 weeks, improved intestinal integrity by increasing the expression of claudin-3, reduced oxidative stress and inflammation, increased beta cell production, resulting in higher insulinemia, and localized mitochondrial dysfunction in the gut [174]. Another study sheds light on the effect of ginger ethanol extract administered via oral gavage for 10 weeks (100 mg/kg/day–200 mg/kg/day) in rats on a high-fat diet, high-carbohydrate diet, showing a reduction in insulin resistance, with an increase in AMPK-α1 in muscles. A similar effect was observed with 6-gingerol, which increases the expression of PGC-1α mRNA, a transcriptional coactivator that regulates cellular energy metabolism, in myoblasts [175].

A factor that significantly influences mitochondrial damage beyond oxidative stress is excess weight [176]. Excessive consumption of processed foods, increasing access to junk food, poor eating habits, and a sedentary lifestyle are strongly associated with the development of obesity, insulin resistance, diabetes, cardiovascular, metabolic, and inflammatory diseases, and cancer, increasing the risk of death [177]. According to the World Health Organization (WHO), obesity-related diseases in developed countries are estimated to reach 30% deaths worldwide by 2030 [178]. The bioactive compounds in ginger appear to be highly functional and nutraceutical, with potential preventive and protective effects against obesity and the frequency of non-communicable diseases.

Interestingly, ginger extract has also been shown to inhibit the transformation of brown adipose tissue into white adipose tissue, thereby protecting tissues from mitochondrial damage by restoring DNA, enzyme activity, and the Uncoupled Protein 1. At the liver and adipose tissue levels, steamed ginger extract activates AMPK-SIRT1, regulating inflammatory signaling processes, reducing hepatic steatosis and metabolic dysfunction in adipocytes and mitochondrial redox potential. (Ginger extract controls mTOR-SREBP1-ER stress-mitochondria dysfunction through AMPK activation in the obesity model).

Epigenetic modifications are closely related to the regulation of the cellular senescence process. Emerging studies suggest that ginger-derived compounds may act as modulators of epigenetic processes. Epigenetic alterations arise from non-genetic mechanisms that regulate gene expression without changes to the underlying DNA sequence, including DNA methylation, histone modifications, and microRNA (miRNA) expression [179,180].

Among these compounds, 6-shogaol has shown promising effects in modulating the activity of histone deacetylases. In cultured primary cardiac fibroblasts, treatment with 6-shogaol (0.2–1 mg/kg for 8 weeks) inhibited the activity of p300 histone acetyltransferase (p300-HAT), resulting in reduced histone H3K9 acetylation in a dose-dependent manner [181]. This effect suppressed the transcriptional activation of genes related to cardiomyocyte hypertrophy and the development of heart failure.

In another study, ginger aqueous extract restored the expression of tumor-suppressive miRNAs in the human breast cancer cell line (MDA-MB-231), including miR-200c, miR-30a, and miR-128, and significantly decreased miR-200C promoter methylation [182].

Considering the available evidence, ginger may represent a promising nutraceutical candidate for healthy aging. Its reported antioxidant, anti-inflammatory, anti-tumoral, and epigenetic regulatory activities enhance further investigation to clarify its potential role in longevity and age-related disease prevention. The goal for a longer lifespan and quality of life lies in promoting health policies focused on prevention and research plans that address this complex phase of life through an integrated approach that also encourages the use of phytochemicals, such as ginger, which, thanks to its nutraceutical value, holds promise for delaying health decline and increasing life expectancy.

### 8.5. Ginger-Gut-Microbiota Interaction and Gastrointestinal Health

Disruption of the physiological function of the gastrointestinal system results in the onset of several acute or chronic digestive disorders, such as dyspepsia, nausea, vomiting, gastric ulcers, and colitis, thereby impairing the quality of life of those affected. Ginger, since ancient times, has been used in many practices of traditional medicine to relieve and treat various pathologies of the gastrointestinal tract and stimulate the production of short-chain fatty acids [183].

In this context, a 12-week randomized trial showed that ginger extract significantly improved abdominal pain, constipation, indigestion, and reflux in patients with functional dyspepsia [184]. Similarly, the clinical trial conducted by Aregawi and colleagues has found that ginger supplementation for two months in patients with functional dyspepsia was well tolerated and could serve as an effective complementary dietary treatment, promoting gastrointestinal health [185]. Cancer treatments such as chemotherapy and radiotherapy are commonly associated with severe gastrointestinal complications. Through its natural antiemetic properties, the administration of ginger to patients with advanced cancer or undergoing chemotherapy has significantly controlled nausea and vomiting, as well as restored appetite [186,187].

Several beneficial effects on gastrointestinal health result from interactions between the bioactive compounds in ginger and the gut microbiota. These effects are mainly attributed to the regulation of inflammation, oxidative stress, and protection of the intestinal epithelial barrier. In an *in vitro* simulation model of gastrointestinal digestion and fermentation, Wang et al. indicated that ginger polyphenols could modulate the gut microbiome by promoting the growth of beneficial bacteria, such as *Bifidobacterium* and *Enterococcus*, which perform biological activities [188]. These beneficial effects were also observed with ginger polysaccharide treatment, which resulted in improved intestinal immune defense in immunosuppressed mice [189].

Gastrointestinal disorders, including ulcerative colitis, Crohn’s disease, and gastric ulcers, are associated with oxidative stress, immune system dysregulation, and microbiota dysbiosis, leading to local and diffuse inflammation, which causes damage to the mucous membranes and alterations in gastrointestinal integrity [190,191,192]. The existing treatments for these illnesses must be administered over an extended period, and their effectiveness is not always guaranteed. They may also cause several adverse effects. This is why research has always focused on finding natural alternative therapies with anti-inflammatory and antioxidant properties [193].

The hydrochloric acid/ethanol experimental model is widely used to study the potential effects of plant extracts on gastric ulceration. The administration of ginger extract reduced lesions and promoted healing of ulcerations in the gastric mucosa by increasing mucus secretion, antioxidant enzymes (SOD and GSH), and PGE2, as well as by reducing lipid peroxidation and myeloperoxidase (MPO) activity [194]. Furthermore, Sistani Karampour et al. demonstrated that the positive effects observed with zingerone treatment were similar to those observed with the reference treatment, ranitidine [195].

In line with the Hao et al. study findings, treatment with ginger polysaccharides alleviated dextran sulphate sodium (DSS)-induced colitis in mouse models experimentally [196]. These effects could be explained by the inhibition of the expression of pro-inflammatory cytokines (TNFα, IL-6, IL-1β, IL-17A, and IFNγ), stimulation of tight junction protein expression (ZO-1 and occludin-1), significant reduction in apoptosis in the colonic epithelium, and modulation of the composition of the intestinal microbiota.

Similarly, Jing Y et al. also showed that these polysaccharides were able to reduce oxidative stress by lowering serum and tissue levels of MDA and MPO and increasing levels of SOD [197]. It has also demonstrated that these bioactive carbohydrate compounds could regulate SCFA levels and control the MyD88/NF-κB/MAPK signaling pathway to downregulate the inflammatory response in colonic tissue. Pretreatment with the bioactive compounds of ginger, shogaols, for two weeks resulted in reduced tissue expression of iNOS and COX2 and modulated expression of mucin 2 (MUC2) and mucin3 (MUC3), thereby attenuating the lesions observed in the colonic epithelial barrier and protecting against induced colitis in mice, as described by Kim et al. [198].

Cancer is a common and serious health problem. Approximately one-third of cancer cases worldwide are attributable to gastrointestinal cancers [199]. Several *in vitro* and *in vivo* studies have also shown that ginger and its natural active ingredients have remarkable antitumor properties. It has been reported that disruption of the cell cycle and stimulation of apoptosis are the key mechanisms involved in inhibiting tumor cell proliferation.

Interestingly, 8-gingerol significantly reduced the viability of gastric (HGC-27) and colorectal (HCT116 and DLD1) cancer cells with IC50 values of 6.2 µM, 77.4 ± 4.70 μM, and 53.7 ± 2.24 μM, respectively. In addition, this compound inhibited the migration and invasion of these two colorectal lines by mediating the EGFR/STAT/ERK signaling pathway [111,200].

The observations of Wang et al. indicated that treatment with zingerone nanoparticles resulted in significant cytotoxicity, enhanced apoptosis, and completely inhibited colony formation in human colon cell lines, LoVo and HCT116, at a concentration of 100 μM [201].

Another active component of ginger, 6-gingerol, has also shown its ability to induce apoptosis in gastric adenocarcinoma cell lines by disrupting mitochondrial homeostasis and stimulating the expression of apoptotic markers, such as caspases, and cytochrome-c [202]. Similar results were obtained by Fayed et al., who demonstrated that ginger extract had remarkable effects in disrupting the homeostasis of Caco-2 cancer cells [203]. The extract inhibited the autophagy mechanism by downregulating the expression of Bectin-1 and Atg5, while simultaneously upregulating proteolytic enzymes, including caspase-3, and tumor suppressor gene PTEN.

Moreover, the treatment with nanoliposomes containing ginger extract for two weeks has reduced tumor size and weight in mice with induced colorectal cancer. In addition, intravenous injections of these nanoliposomes significantly stimulated expression of the pro-apoptotic gene Bax, resulting in a major antiproliferative effect [204].

All these findings suggest that ginger and its phytochemical derivatives have relevant pharmacological properties, including anti-inflammatory, antioxidant, immunomodulatory, and anticancer activities, which may constitute a potential therapeutic strategy against several digestive system disorders, particularly gastrointestinal ulcers and cancers (Figure 2).

Table 3 summarizes the pleiotropic roles of the main bioactive compounds present in ginger, highlighting their different biological activities, including anti-inflammatory, antioxidant, cardiovascular protective, metabolic regulatory, and neuroprotective effects.

## 9. AI Applications in Ginger Research

A rapidly emerging research area focuses on bioactive molecules and their pleiotropic effects, investigating both their therapeutic potential and broader biological impacts. The integration of machine learning (ML) and artificial intelligence (AI) enhances this field by supporting the prediction of molecular interactions, identification of therapeutic targets, and modelling of multi-pathway mechanisms of action [207,208]. Interestingly, AI-based computational approaches can facilitate the discovery of bioactive agents with therapeutic relevance across diverse biological systems. For example, AI-driven peptide discovery has revealed the potential of deep learning architectures such as convolutional neural networks, long short-term memory networks, and Transformer models to design and optimize functional bioactive sequences [209].

This provides a solid methodological framework for applying similar strategies to the study of *Zingiber officinale*. Using AI and ML techniques can thus enhance the identification, characterization, and mechanistic analysis of ginger-derived phytochemicals. Data-driven approaches, including deep learning, network pharmacology, and systems biology, enable the integration of multi-omics and molecular interaction data to examine relationships between ginger constituents and human molecular networks. Computational modelling further predicts compound–target interactions and associated signaling pathways underlying reported anti-inflammatory, antioxidant, and anticancer effects [210,211].

Furthermore, modern AI models facilitate predictive modelling, compound–target interaction mapping, and mechanistic pathway analysis, effectively repositioning ginger from a traditional herbal remedy to a data-driven resource for drug development [212]. Recent advances in deep learning, network pharmacology, and systems biology, particularly in model interpretability and multimodal integration, have revealed previously unrecognized connections between key ginger’s bioactive compounds, such as 6-gingerol and 6-shogaol, and diverse human protein targets. Together, these approaches integrate computational innovation with biological insight to uncover molecular mechanisms and support systematic natural product-based drug discovery [213,214,215].

This integration of AI with biochemical systems research represents a paradigm shift toward a more precise, predictive, and holistic understanding of natural products such as ginger in drug discovery and translational medicine. Deep learning and systems biology approaches support molecular docking, metabolic pathway analysis, and pharmacokinetic modelling, enabling identification of synergistic interactions between ginger’s bioactive compounds and key signaling pathways, including NF-κB, PI3K/Akt, and MAPK, which are central to inflammation and cancer biology.

Furthermore, this fusion of computational intelligence with biochemical research accelerates not only the discovery of novel therapeutic targets but also the visualization of complex multi-pathway interactions, elucidating the pleiotropic effects characteristic of ginger phytochemicals [216]. Taken together, these advances highlight increasing convergence of traditional phytotherapy and computational drug discovery, marking the beginning of a new era in natural product pharmacology.

### 9.1. Predictive Modelling for Bioactive Compound Interactions with Molecular Targets: Focus on Ginger

As discussed earlier, ginger contains diverse bioactive compounds, including 6-gingerol, 6-shogaol, zingerone, paradols, and gingerenone-A, which underpin its broad pharmacological activities [35]. These constituents exert anti-inflammatory and antioxidant effects by modulating key signaling pathways such as NF-κB, MAPK, PI3K/Akt/mTOR, AMPK, and Nrf2. Through this coordinated regulation, they suppress pro-inflammatory mediators (NO, COX-2, PGE_2_, TNFα, IL-1β), inhibit NLRP3 inflammasome activation, and attenuate oxidative stress in macrophages and neutrophils. Notably, 6-gingerol activates Nrf2-dependent antioxidant responses while concurrently suppressing NF-κB signaling and downstream cytokines such as TNFα and IL-6 [11]. Collectively, these findings highlight the pleiotropic, multi-target actions of ginger bioactives, which generate synergistic anti-inflammatory, cardioprotective, and metabolic-regulatory effects [104,217].

This intricate crosstalk between cellular pathways highlights the sophisticated pharmacodynamics of ginger, necessitating the use of predictive modelling tools to dissect its pleiotropic mechanisms. Predictive modelling has emerged as a critical tool in understanding how bioactive compounds interact with molecular targets. Advances in computational biology have enabled predictive modelling approaches, such as molecular docking, molecular dynamics (MD) simulations, network pharmacology, and machine-learning algorithms to explore multitarget interactions of ginger bioactives [123,210,218,219].

Docking analyses identify binding modes and affinities between compounds and protein targets, while MD assesses complex stability. Gingerenone-A and shogaol showed strong binding to Staphylococcus aureus HPPK, a key enzyme in folate biosynthesis, and MD simulations confirmed the structural stability of these complexes [206]. In parallel, network pharmacology analysis linked six major ginger compounds to 285 colon cancer-related targets, identifying 34 key genes—including TP53, HSP90AA1, and JAK2—identified through PPI network and molecular docking validation. These targets were mainly enriched in PI3K signaling, oxidative stress response, and endocrine resistance pathways, highlighting ginger’s multi-component, multi-target mechanism in colon cancer prevention [220]. Similarly, antioxidant and immunomodulatory activities of ginger leaf compounds were revealed through integrated network pharmacology and experimental validation [36].

The biological effects of ginger are largely synergistic, arising from the cooperative actions of multiple phytochemicals on shared molecular targets. Advanced modelling techniques, including multi-ligand docking and network-based synergy prediction, enable systematic discrimination between additive and synergistic interactions. Multi-target virtual screening has identified compound combinations with enhanced inhibitory potential against cancer-associated signaling pathways [221,222]. Consistent with these predictions, combined in silico and *in vitro* studies demonstrate that 6-gingerol and 6-shogaol exhibit complementary binding to inflammatory mediators such as COX-2, iNOS, and HDAC8, producing greater suppression of TNFα, IL-6, and NO than individual treatments [223,224,225]. Together, these approaches connect computational modelling with experimental validation, facilitating target prioritization and rational optimization of multi-component ginger-derived therapeutics for modern drug discovery applications.

### 9.2. AI-Driven Predictive Modelling and Target Discovery in Ginger Research

Recent advances in AI–driven quantitative structure–activity relationship (QSAR) modelling and deep learning frameworks have greatly enhanced our ability to predict compound–target interactions [226,227]. In ginger research, these approaches have been applied to characterize the binding potential of major metabolites such as 6-gingerol and 6-shogaol toward inflammation- and oxidative stress-related targets [228].

Emerging architectures such as Graph Neural Networks (GNNs) and Transformer-based SMILES encoders can effectively represent molecular graph topology and electronic environments, enabling improved prediction of ligand–target affinity prediction [212]. In ginger-focused studies, docking combined with ML-based rescoring has confirmed favorable interactions of gingerol derivatives with COX-2, NF-κB-related proteins, and TRPV1 key mediators of inflammation and pain [205,229,230,231,232]. These hybrid AI–docking pipelines allow prioritization of high-confidence candidates for experimental validation, improving screening efficiency compared with docking alone. Beyond target affinity prediction, AI-powered in silico systems facilitate multi-omics integration, combining metabolomic, transcriptomic, and proteomic datasets to map complex regulatory networks implicated in metabolic, oncologic, and infectious diseases [233,234,235].

Moreover, target fishing algorithms and reverse docking methods supported by ML enable the discovery of previously unrecognized protein partners for ginger compounds, while ADMET (Absorption, Distribution, Metabolism, Excretion, and Toxicity) predictors enhance preclinical safety evaluation. Explainable AI (XAI) approaches such as SHAP and LIME provide interpretability to these models, helping visualize how molecular substructures contribute to multi-target binding and biological efficacy [236,237].

Finally, AI-driven network pharmacology and deep-docking reinforcement learning are exploring novel targets for ginger’s bioactive ingredients, including PPARγ, EGFR, NLRP3 inflammasome, and BDNF molecules linked to metabolic regulation, renal protection, and neuroprotection [1,123,214,238]. Collectively, these computational strategies position ginger within a data-driven framework for precision natural product drug discovery.

### 9.3. Data Visualization: Network Mapping of Pleiotropic Effects in Ginger

AI-driven visualization frameworks have been applied to enhance our understanding of the pleiotropic mechanisms underlying ginger. Network pharmacology combined with molecular docking has demonstrated that 6-gingerol regulates multiple targets involved in lipid metabolism and atherosclerosis-related pathways [239]. While methods such as Cytoscape are commonly used for compound–target visualization in natural-product research, there remains little published evidence of embedding techniques (e.g., DeepWalk) or explainable-AI (SHAP, LIME) analyses applied directly to ginger metabolite networks. To date, systematic reviews of AI in pharmacology focus on drug–drug and drug-nutrient interactions broadly and do not report specific feature-interpretation studies of ginger compounds [240].

Hence, the integration of deep-learning embeddings, ontology-driven networks, and XAI for exploring ginger’s multi-target interactions remains an emergent, rather than fully realized, area of research. Collectively, these visualization-driven insights advance a systems-level understanding of ginger’s pharmacodynamics, transforming it from a traditional herbal remedy into a computationally decoded therapeutic network with relevance across inflammation, neuroprotection, and metabolic regulation.

## 10. Conclusions and Future Directions

From this narrative review, it emerges that the bioactive compounds of ginger, in particular, gingerols and shogaols, offer therapeutic potential for cardiovascular, neurodegenerative, arthritis, and gastrointestinal diseases through their anti-inflammatory, antimicrobial, and antioxidant properties, through the modulation of signaling pathways.

Moreover, ginger appears to be a good nutraceutical for longevity, delaying health decline and increasing life expectancy; in fact, the recent literature reveals an anti-aging activity of ginger, which can mitigate age-related cellular damage, a key factor in aging and age-related diseases.

It follows that the bioactive molecules in ginger can promote healthy aging by reducing morbidity and extending a healthy lifespan.

Therefore, ginger shows multi-system beneficial effects with potential for preventive and therapeutic use. The mechanisms and effects of ginger, namely, the role of ginger in promoting overall well-being and disease prevention, are elevated, leading to the consideration of ginger root as a valuable component in integrative health practices.

AI can also accelerate the discovery of new bioactive compounds with therapeutic relevance. By analyzing large datasets, it can be of valid support in the identification of bioactive compounds present in ginger with new mechanisms of action.

Integration of AI tools can enhance literature analysis and identify novel research directions. This study provides a holistic, modern perspective, bridging traditional medicine and computational approaches.

Importantly, these approaches may allow for the identification of novel molecular targets, opening avenues for new potential clinical applications for ginger and accelerating translational research in natural product-based therapeutics, with particular focus on ginger’s bioactive ingredients in various pathological contexts. The use of AI could be of valid help in choosing the bioactive compounds of ginger, for example, whether to use fresh or dried ginger, or to use one type of extraction instead of another, which would involve an enrichment of some compounds instead of others in relation to the pathological condition to be treated and therefore to the therapeutic target identified. This approach would be of great use for targeted treatments and precision medicine.

Taken together, these findings highlight the multifaceted potential of ginger as a valuable ingredient for integration into functional food and nutraceutical products.

Continued interdisciplinary research is warranted to substantiate its therapeutic efficacy and ensure long-term safety in human applications.

## Figures and Tables

**Table 1:** Nanoformulations of ginger for bioavailability enhancement.

Formulation	Experimental Model	Bioavailability Improvement	Outcome	Ref.
**Chitosan nanoparticles loaded with** ***Zingiber officinale* essential oil**	*In vitro* Physicochemical characterization Agar diffusion assay	Positive surface charge → enhanced mucoadhesion and interaction with mucus layer	Improved antimicrobial activity and delivery potential of bioactive compounds	[66]
**Chitosan-coated** **6-gingerol nanoemulsion (intranasal)**	*In vitro* (release, stability) Ex vivo (nasal permeation)	Increased mucoadhesion and nasal permeation → enhanced brain-targeted delivery	Improved brain bioavailability and barrier permeation (BBB)	[66,67]
**Phytosomal ginger extract** **(±*Rosa canina* L.)**	*In vivo* C57BL/6J mice (LPS-induced inflammation)	2.6-fold increase in plasma 6-gingerol >40% increase in liver/kidney 65% decrease in gastric levels vs. non-formulated extract	Enhanced antioxidant and anti-inflammatory effects Improved systemic distribution	[68]
**Nanoliposomal 6-gingerol**	*In vitro* PDL fibroblasts MDA-MB-231 cells	Improved physicochemical stability (30 days at 4 °C) Preserved biological activity	Enhanced anti-proliferative activity and IL-8 inhibition	[71]
**Ginger-derived edible nanoparticles** **(6-gingerol; 6-shogaol)**	*In vivo* (mouse colitis models)	Efficient colon targeting after oral administration High biocompatibility Reduced systemic toxicity	Colon-specific accumulation therapeutic benefit in inflammatory models	[72]

**Table 2:** Ginger in arthritis and musculoskeletal health.

Formulation/ Compound	Experimental Model	Main Outcome/Key Pathways	Ref.
***In vitro* models**			
**Ginger extract + *Alpinia galanga* extract**	Human synoviocytes activated with TNFα (1 ng/mL)	Suppressed expression of chemokines, MCP-1 and IP-10	[137]
**Zingerone**	Arthritic fibroblast-like synoviocytes (FLSs) stimulated with TNFα	Suppressed TNFα-induced proliferation, migration, ROS formation, and transcript levels of IL-1β and IL-6 by targeting MAPKs (ERK, p38, JNK) signaling.	[138]
**α-Cedrol**	FLSs exposed to LPS (*in vitro*)	Reduction in ERK/MAPK and p65 NF-κB activation; reduced expression of COX-1/COX-2 (mRNA and protein), inhibited PGE2 release, reduced TNFα and IL-1β, and reduced MMP-13 and MCP-1 mRNA.	[139]
**6-Shogaol**	Human rheumatoid FLS line MH7A and RA FLSs	Inhibited proliferation, migration, and invasion; induced apoptosis; reduced TNFα, IL-1β, IL-6, IL-8, MMP-2, MMP-9; inhibited PI3K/AKT/NF-κB signaling via PPAR-γ activation.	[140]
**Ginger extract**	C28/I2 human chondrocyte cell line activated with IL-1β	Increased gene expression of antioxidant enzymes; reduced IL-1β-induced ROS, lipid peroxidation, Bax/Bcl-2 ratio, and caspase-3 activity, with no evident cell toxicity.	[141]
**Ginger extract**	Chondrocytes from normal and osteoarthritic sow cartilage explants under cytokine stimulation (24 h)	Reduced NO and PGE2 production.	[142]
**6-Shogaol**	Chondrocyte *in vitro* models (LPS/TLR4 induction), ATDC5 murine chondrogenic cell line, and human primary chondrocytes	Inhibition NO production; reduced IL-6 and MCP-1; reduced ERK1/2 phosphorylation along with NOS2 and MyD88 downregulation; repressed MMP2/MMP9 activity; reduced cathepsin-K activity in human primary chondrocytes.	[143]
**Aqueous *Zingiber officinale* root extract (HPLC: 1,4-cineol, 6-gingerol, vanillylacetone, shogaol as main molecules)**	Human synovial fibroblasts activated by IL-1β	Decreased IFNγ, IL-17, MMP-3, MMP-13 mRNA expression.	[145]
***In vivo* models**			
**α-Cedrol**	DBA/1J mice with collagen-induced arthritis (CIA)	Attenuated severity of CIA pathology.	[139]
**6-Shogaol**	Mice with CIA	Attenuated joint destruction.	[140]
**Hydroalcoholic *Zingiber officinale* rhizome extract**	Rat CIA model treated with a dose of 50, 100, and 200 mg/kg daily by intraperitoneal injection	Improved clinical scores, disease incidence, joint temperature and swelling, and cartilage destruction; reduced serum IL-6, TNFα, IL-1β, IL-2, and anti-CII antibodies.	[144]
**Aqueous *Zingiber officinale* root extract (HPLC: 1,4-cineol, 6-gingerol, vanillylacetone, shogaol as main molecules)**	Mice with CIA treated daily by oral administration	Decreased serum level of IL-4, IFNγ, IL-17; inhibited IL-17 expression in spleen and paw tissues; downregulated MMP-1, MMP-3, MMP-13 in paw tissues; reduced inflammatory bone damage within joints.	[145]
**Ginger root extract**	Rat CIA model treated daily by oral administration (50 mg/kg/day)	Decreased serum TNFα, IL-6, IL-17, DKK-1. Increased serum sclerostin. Reduced tissue IL-17, NF-κB;	[45]
**Dehydrozingerone (DHZ)**	CFA-induced arthritis rat model. Intragastrical administration 100 mg/kg.	Attenuated disease severity: reduced global arthritic score. Reduced serum ALP, AST/ALT, rheumatoid factor, CRP, TNFα, TGFβ, IL-1β, IL-6, malondialdehyde and VEGF; Increased serum SOD and GSH.	[146]
***Clinical trials and Human Evidence***			
**Powdered *Zingiber officinale***	Randomized controlled trial; patients with knee osteoarthritis. Treatment: 500 mg of powdered ginger twice daily for 3 months	Reduced serum TNFα and IL-1β levels.	[147]
**Oral ginger**	Meta-analysis in osteoarthritis (OA) symptomatic treatment	Modest significant reduction in pain and disability; higher likelihood of discontinuation due to adverse events.	[148]
**Steamed ginger extract (GGE03)**	Randomized controlled trial; Treatment: 1600 mg of GGE03/day in mild knee osteoarthritis	Statistically significant improvements in pain, stiffness, function, and patient global assessment vs. placebo; no adverse safety events observed.	[149]
**Ginger extract standardized to 10% total gingerols and ≤3% total shogaols**	Randomized, placebo-controlled trial; Treatment: 125 mg/d of ginger extract in exercise-induced mild-to-moderate muscle and joint pain	Favorable effects on exercise-induced perceptions of pain, functional capacity, and inflammatory markers.	[150]
**Ginger extract standardized to 10% total gingerols and ≤3% total shogaols**	Randomized, placebo-controlled trial; Treatment: 125 mg/d of ginger extract in exercise-induced mild-to-moderate muscle and joint pain	Favorable effects on knee range of motion, markers of health, and perceptions about quality of life.	[151]

**Table 3:** Pleiotropic role of *Zingiber officinale*.

Bioactive Compound	Function	Localization/Type	Ref.
**6-Gingerol**	Antioxidant, anti-inflammatory, anti-apoptotic; renal, hepatic, and pulmonary protection; modulation of NF-κB/PI3K-Akt pathways, antibacterial	Phenolic; most abundant in fresh ginger	[1,107,109,110,111,117]
**10-Gingerol**	Antioxidant and anti-inflammatory	Phenolic	[11,112]
**6-Shogaol**	Strong anti-inflammatory, anticancer, antioxidant; epigenetic effects; ROS/MAPK modulation, neuroprotective effects, antibacterial	Formed from gingerols by dehydration/heat	[11,34,39,106,109,111,118,127]
**Zingerone**	Antioxidant, anticancer, pro-apoptotic	Phenolic (formed in dried ginger)	[47,52,57,138,158,195,201]
**6-Paradol**	Antibacterial	Phenolic	[32,135,205]
**Zerumbone**	Anti-inflammatory, anticancer, anti-angiogenic, antioxidant, anti-microbial	Sesquiterpene	[29,30]
**Gingerenone-A**	Anti-inflammatory; NF-κB/PI3K-mTOR modulation	Phenolic	[171,206]
**Polysaccharides, flavonoids, lipids, organic acids, and dietary fiber**	anti-obesity, antidiabetic, antioxidant and cardiovascular protective effects	Nutritional value	[2,35]

## Data Availability

The original contributions presented in this study are included in the article. Further inquiries can be directed to the corresponding author.

## References

[1] Almatroodi S.A., Alnuqaydan A.M., Babiker A.Y., Almogbel M.A., Khan A.A., Husain Rahmani A. (2021). 6-Gingerol, a Bioactive Compound of Ginger Attenuates Renal Damage in Streptozotocin-Induced Diabetic Rats by Regulating the Oxidative Stress and Inflammation. Pharmaceutics.

[2] Edo G.I., Igbuku U.A., Makia R.S., Isoje E.F., Gaaz T.S., Yousif E., Jikah A.N., Zainulabdeen K., Akpoghelie P.O., Opiti R.A. (2025). Phytochemical Profile, Therapeutic Potentials, Nutritional Composition, and Food Applications of Ginger: A Comprehensive Review. Discov. Food.

[3] Li C., Li J., Jiang F., Tzvetkov N.T., Horbanczuk J.O., Li Y., Atanasov A.G., Wang D. (2021). Vasculoprotective Effects of Ginger (*Zingiber officinale* Roscoe) and Underlying Molecular Mechanisms. Food Funct..

[4] Ajanaku C.O., Ademosun O.T., Atohengbe P.O., Ajayi S.O., Obafemi Y.D., Owolabi O.A., Akinduti P.A., Ajanaku K.O. (2022). Functional Bioactive Compounds in Ginger, Turmeric, and Garlic. Front. Nutr..

[5] Ayustaningwarno F., Anjani G., Ayu A.M., Fogliano V. (2024). A Critical Review of Ginger’s (*Zingiber officinale*) Antioxidant, Anti-Inflammatory, and Immunomodulatory Activities. Front. Nutr..

[6] Park E.J., Pezzuto J.M. (2002). Botanicals in Cancer Chemoprevention. Cancer Metastasis Rev..

[7] Shaukat M.N., Nazir A., Fallico B. (2023). Ginger Bioactives: A Comprehensive Review of Health Benefits and Potential Food Applications. Antioxidants.

[8] Hu W., Yu A., Wang S., Bai Q., Tang H., Yang B., Wang M., Kuang H. (2023). Extraction, Purification, Structural Characteristics, Biological Activities, and Applications of the Polysaccharides from *Zingiber officinale* Roscoe. (Ginger): A Review. Molecules.

[9] Nair K.P. (2019). Turmeric (Curcuma longa L.) and Ginger (Zingiber officinale Rosc.)—World’s Invaluable Medicinal Spices: The Agronomy and Economy of Turmeric and Ginger.

[10] Arcusa R., Villaño D., Marhuenda J., Cano M., Cerdà B., Zafrilla P. (2022). Potential Role of Ginger (*Zingiber officinale* Roscoe) in the Prevention of Neurodegenerative Diseases. Front. Nutr..

[11] Pázmándi K., Szöllősi A.G., Fekete T. (2024). The “Root” Causes behind the Anti-Inflammatory Actions of Ginger Compounds in Immune Cells. Front. Immunol..

[12] Dalsasso R.R., Valencia G.A., Monteiro A.R. (2022). Impact of Drying and Extractions Processes on the Recovery of Gingerols and Shogaols, the Main Bioactive Compounds of Ginger. Food Res. Int..

[13] Mahboubi M. (2019). *Zingiber officinale* Rosc. Essential Oil, a Review on Its Composition and Bioactivity. Clin. Phytosci..

[14] Jabborova D., Choudhary R., Azimov A., Jabbarov Z., Selim S., Abu-Elghait M., Desouky S.E., Azab I.H.E., Alsuhaibani A.M., Khattab A. (2022). Composition of *Zingiber officinale* Roscoe (Ginger), Soil Properties and Soil Enzyme Activities Grown in Different Concentration of Mineral Fertilizers. Horticulturae.

[15] Jung M.Y., Lee M.K., Park H.J., Oh E.-B., Shin J.Y., Park J.S., Jung S.Y., Oh J.-H., Choi D.-S. (2018). Heat-Induced Conversion of Gingerols to Shogaols in Ginger as Affected by Heat Type (Dry or Moist Heat), Sample Type (Fresh or Dried), Temperature and Time. Food Sci. Biotechnol..

[16] Sang S., Snook H.D., Tareq F.S., Fasina Y. (2020). Precision Research on Ginger: The Type of Ginger Matters. J. Agric. Food Chem..

[17] Roudsari N.M., Lashgari N.-A., Momtaz S., Roufogalis B., Abdolghaffari A.H., Sahebkar A. (2021). Ginger: A Complementary Approach for Management of Cardiovascular Diseases. BioFactors.

[18] Schepici G., Contestabile V., Valeri A., Mazzon E. (2021). Ginger, a Possible Candidate for the Treatment of Dementias?. Molecules.

[19] Ahmed S.H.H., Gonda T., Agbadua O.G., Girst G., Berkecz R., Kúsz N., Tsai M.-C., Wu C.-C., Balogh G.T., Hunyadi A. (2023). Preparation and Evaluation of 6-Gingerol Derivatives as Novel Antioxidants and Antiplatelet Agents. Antioxidants.

[20] Sharma S., Shukla M.K., Sharma K.C., Tirath, Kumar L., Anal J.M.H., Upadhyay S.K., Bhattacharyya S., Kumar D. (2023). Revisiting the Therapeutic Potential of Gingerols against Different Pharmacological Activities. Naunyn-Schmiedebergs Arch. Pharmacol..

[21] Zhang C., Rao A., Chen C., Li Y., Tan X., Long J., Wang X., Cai J., Huang J., Luo H. (2024). Pharmacological Activity and Clinical Application Analysis of Traditional Chinese Medicine Ginger from the Perspective of One Source and Multiple Substances. Chin. Med..

[22] Anh N.H., Kim S.J., Long N.P., Min J.E., Yoon Y.C., Lee E.G., Kim M., Kim T.J., Yang Y.Y., Son E.Y. (2020). Ginger on Human Health: A Comprehensive Systematic Review of 109 Randomized Controlled Trials. Nutrients.

[23] Yusuf A.A., Lawal B., Abubakar A.N., Berinyuy E.B., Omonije Y.O., Umar S.I., Shebe M.N., Alhaji Y.M. (2018). In-Vitro Antioxidants, Antimicrobial and Toxicological Evaluation of Nigerian *Zingiber officinale*. Clin. Phytosci..

[24] Krüger S., Bergin A., Morlock G.E. (2018). Effect-Directed Analysis of Ginger (*Zingiber officinale*) and Its Food Products, and Quantification of Bioactive Compounds via High-Performance Thin-Layer Chromatography and Mass Spectrometry. Food Chem..

[25] Hu J., Guo Z., Glasius M., Kristensen K., Xiao L., Xu X. (2011). Pressurized Liquid Extraction of Ginger (*Zingiber officinale* Roscoe) with Bioethanol: An Efficient and Sustainable Approach. J. Chromatogr. A.

[26] Ghasemzadeh A., Jaafar H.Z.E., Rahmat A. (2015). Optimization Protocol for the Extraction of 6-Gingerol and 6-Shogaol from *Zingiber officinale* Var. Rubrum Theilade and Improving Antioxidant and Anticancer Activity Using Response Surface Methodology. BMC Complement. Altern. Med..

[27] Maghraby Y.R., Labib R.M., Sobeh M., Farag M.A. (2023). Gingerols and Shogaols: A Multi-Faceted Review of Their Extraction, Formulation, and Analysis in Drugs and Biofluids to Maximize Their Nutraceutical and Pharmaceutical Applications. Food Chem. X.

[28] Ghasemzadeh A., Jaafar H.Z.E., Baghdadi A., Tayebi-Meigooni A. (2018). Formation of 6-, 8- and 10-Shogaol in Ginger through Application of Different Drying Methods: Altered Antioxidant and Antimicrobial Activity. Molecules.

[29] Kim A., Gwon M.-H., Lee W., Moon H.-R., Yun J.-M. (2022). Zerumbone Suppresses High Glucose and LPS-Induced Inflammation in THP-1-Derived Macrophages by Inhibiting the NF-κB/TLR Signaling Pathway. Nutr. Res..

[30] Moreira Da Silva T., Pinheiro C.D., Puccinelli Orlandi P., Pinheiro C.C., Soares Pontes G. (2018). Zerumbone from *Zingiber zerumbet* (L.) Smith: A Potential Prophylactic and Therapeutic Agent against the Cariogenic Bacterium *Streptococcus* Mutans. BMC Complement. Altern. Med..

[31] Assiry A.A., Ahmed N., Almuaddi A., Saif A., Alshahrani M.A., Mohamed R.N., Karobari M.I. (2023). The Antioxidant Activity, Preliminary Phytochemical Screening of *Zingiber zerumbet* and Antimicrobial Efficacy against Selective Endodontic Bacteria. Food Sci. Nutr..

[32] Rahmani A.H., Shabrmi F.M.A., Aly S.M. (2014). Active Ingredients of Ginger as Potential Candidates in the Prevention and Treatment of Diseases via Modulation of Biological Activities. Int. J. Physiol. Pathophysiol. Pharmacol..

[33] Sulieman A.M.E., Ibrahim S.M., Alshammari M., Abdulaziz F., Idriss H., Alanazi N.A.H., Abdallah E.M., Siddiqui A.J., Shommo S.A.M., Jamal A. (2024). *Zingiber officinale* Uncovered: Integrating Experimental and Computational Approaches to Antibacterial and Phytochemical Profiling. Pharmaceuticals.

[34] Yang C., Chen W., Ye B., Nie K. (2024). An Overview of 6-Shogaol: New Insights into Its Pharmacological Properties and Potential Therapeutic Activities. Food Funct..

[35] Plana L., Marhuenda J., Arcusa R., García-Muñoz A.M., Ballester P., Cerdá B., Victoria-Montesinos D., Zafrilla P. (2025). Characterization, Antioxidant Capacity, and In Vitro Bioaccessibility of Ginger (*Zingiber officinale* Roscoe) in Different Pharmaceutical Formulations. Antioxidants.

[36] Nam D.-G., Kim M., Choi A.-J., Choe J.-S. (2024). Health Benefits of Antioxidant Bioactive Compounds in Ginger (*Zingiber officinale*) Leaves by Network Pharmacology Analysis Combined with Experimental Validation. Antioxidants.

[37] Ballester P., Cerdá B., Arcusa R., Marhuenda J., Yamedjeu K., Zafrilla P. (2022). Effect of Ginger on Inflammatory Diseases. Molecules.

[38] Ghasemzadeh A., Jaafar H.Z.E., Rahmat A. (2010). Antioxidant Activities, Total Phenolics and Flavonoids Content in Two Varieties of Malaysia Young Ginger (*Zingiber officinale* Roscoe). Molecules.

[39] Bak M.-J., Ok S., Jun M., Jeong W.-S. (2012). 6-Shogaol-Rich Extract from Ginger up-Regulates the Antioxidant Defense Systems in Cells and Mice. Molecules.

[40] Carnuta M.G., Deleanu M., Barbalata T., Toma L., Raileanu M., Sima A.V., Stancu C.S. (2018). *Zingiber officinale* Extract Administration Diminishes Steroyl-CoA Desaturase Gene Expression and Activity in Hyperlipidemic Hamster Liver by Reducing the Oxidative and Endoplasmic Reticulum Stress. Phytomedicine.

[41] Idris N.A., Yasin H.M., Usman A. (2019). Voltammetric and Spectroscopic Determination of Polyphenols and Antioxidants in Ginger (*Zingiber officinale* Roscoe). Heliyon.

[42] Ahmed N., Karobari M.I., Yousaf A., Mohamed R.N., Arshad S., Basheer S.N., Peeran S.W., Noorani T.Y., Assiry A.A., Alharbi A.S. (2022). The Antimicrobial Efficacy Against Selective Oral Microbes, Antioxidant Activity and Preliminary Phytochemical Screening of *Zingiber officinale*. Infect. Drug Resist..

[43] Algandaby M.M., El-halawany A.M., Abdallah H.M., Alahdal A.M., Nagy A.A., Ashour O.M., Abdel-Naim A.B. (2016). Gingerol Protects against Experimental Liver Fibrosis in Rats via Suppression of Pro-Inflammatory and Profibrogenic Mediators. Naunyn-Schmiedebergs Arch. Pharmacol..

[44] Bischoff-Kont I., Fürst R. (2021). Benefits of Ginger and Its Constituent 6-Shogaol in Inhibiting Inflammatory Processes. Pharmaceuticals.

[45] Öz B., Orhan C., Tuzcu M., Şahin N., Özercan İ.H., Demirel Öner P., Koca S.S., Juturu V., Şahin K. (2021). Ginger Extract Suppresses the Activations of NF-κB and Wnt Pathways and Protects Inflammatory Arthritis. Eur. J. Rheumatol..

[46] Aryaeian N., Mahmoudi M., Shahram F., Poursani S., Jamshidi F., Tavakoli H. (2019). The Effect of Ginger Supplementation on IL2, TNFα, and IL1β Cytokines Gene Expression Levels in Patients with Active Rheumatoid Arthritis: A Randomized Controlled Trial. Med. J. Islam. Repub. Iran.

[47] Hafezizadeh M., Salehcheh M., Mohtadi S., Mansouri E., Khodayar M.J. (2024). Zingerone Effects on Arsenic-Induced Glucose Intolerance and Hepatotoxicity in Mice via Suppression of Oxidative Stress-Mediated Hepatic Inflammation and Apoptosis. J. Trace Elem. Med. Biol..

[48] Gabr S.A., Alghadir A.H., Ghoniem G.A. (2019). Biological Activities of Ginger against Cadmium-Induced Renal Toxicity. Saudi J. Biol. Sci..

[49] Ayinla M.T., Asuku A.O. (2025). The Neurotoxic Effects of Lead Acetate and the Abrogating Actions of 6-Gingerol-Rich Extract of Ginger via Modulation of Antioxidant Defence System, pro-Inflammatory Markers, and Apoptotic Cascade. Naunyn-Schmiedebergs Arch. Pharmacol..

[50] Widowati W., Kusuma H.S.W., Priyandoko D., Surakusumah W., Dewi N.S.M., Rahmat D., Laksmitawati D.R., Pratami D.K., Azis R., Hadiprasetyo D.S. (2025). Hepatoprotective Activity of Red Ginger Extract on Acetaminophen-induced Liver Injury Models in HepG2 Cells. Ann. Afr. Med..

[51] Wang Y., Jiang Y., Han C., Zhou L., Hu H., Song H., Li W. (2025). Ginger (*Zingiber officinale* Roscoe) Bioactive Components: Potential Resources for Kidney Health. J. Food Biochem..

[52] Kandemir F.M., Yildirim S., Kucukler S., Caglayan C., Mahamadu A., Dortbudak M.B. (2018). Therapeutic Efficacy of Zingerone against Vancomycin-Induced Oxidative Stress, Inflammation, Apoptosis and Aquaporin 1 Permeability in Rat Kidney. Biomed. Pharmacother..

[53] Imani H., Tabibi H., Najafi I., Atabak S., Hedayati M., Rahmani L. (2015). Effects of Ginger on Serum Glucose, Advanced Glycation End Products, and Inflammation in Peritoneal Dialysis Patients. Nutr. Burbank Los Angel. Cty. Calif..

[54] Al Syaad K.M., Elsaid F.G., Abdraboh M.E., Al-Doaiss A.A. (2019). Effect of Graviola (*Annona muricata* L.) and Ginger (*Zingiber officinale* Roscoe) on Diabetes Mellitus Induced in Male Wistar Albino Rats. Folia Biol..

[55] Han H.S., Kim K.B., Jung J.H., An I.S., Kim Y.-J., An S. (2018). Anti-Apoptotic, Antioxidant and Anti-Aging Effects of 6-Shogaol on Human Dermal Fibroblasts. Biomed. Dermatol..

[56] Wang Y.K., Hong Y.J., Yao Y.H., Huang X.M., Liu X.B., Zhang C.Y., Zhang L., Xu X.L. (2013). 6-Shogaol Protects against Oxidized LDL-Induced Endothelial Injruries by Inhibiting Oxidized LDL-Evoked LOX-1 Signaling. Evid.-Based Complement. Altern. Med. ECAM.

[57] Rashid S., Wali A.F., Rashid S.M., Alsaffar R.M., Ahmad A., Jan B.L., Paray B.A., Alqahtani S.M.A., Arafah A., Rehman M.U. (2021). Zingerone Targets Status Epilepticus by Blocking Hippocampal Neurodegeneration via Regulation of Redox Imbalance, Inflammation and Apoptosis. Pharmaceuticals.

[58] Sp N., Kang D.Y., Lee J.-M., Bae S.W., Jang K.-J. (2021). Potential Antitumor Effects of 6-Gingerol in P53-Dependent Mitochondrial Apoptosis and Inhibition of Tumor Sphere Formation in Breast Cancer Cells. Int. J. Mol. Sci..

[59] Figueroa-González G., Quintas-Granados L.I., Reyes-Hernández O.D., Caballero-Florán I.H., Peña-Corona S.I., Cortés H., Leyva-Gómez G., Habtemariam S., Sharifi-Rad J. (2024). Review of the Anticancer Properties of 6-Shogaol: Mechanisms of Action in Cancer Cells and Future Research Opportunities. Food Sci. Nutr..

[60] Mustafa I., Chin N.L. (2023). Antioxidant Properties of Dried Ginger (*Zingiber officinale* Roscoe) Var. Bentong. Foods.

[61] Cui C., Du M., Zhao Y., Tang J., Liu M., Min G., Chen R., Zhang Q., Sun Z., Weng H. (2024). Functional Ginger-Derived Extracellular Vesicles-Coated ZIF-8 Containing TNF-α siRNA for Ulcerative Colitis Therapy by Modulating Gut Microbiota. ACS Appl. Mater. Interfaces.

[62] Wang X., Zhang D., Jiang H., Zhang S., Pang X., Gao S., Zhang H., Zhang S., Xiao Q., Chen L. (2020). Gut Microbiota Variation with Short-Term Intake of Ginger Juice on Human Health. Front. Microbiol..

[63] Bode A.M., Dong Z., Benzie I.F.F., Wachtel-Galor S. (2011). The Amazing and Mighty Ginger. Herbal Medicine: Biomolecular and Clinical Aspects.

[64] Hazra S., Singh P.A. (2024). Safety Aspects of Herb Interactions: Current Understanding and Future Prospects. Curr. Drug Metab..

[65] Gupta J., Kumar D., Gupta R., Kumar S., Kumar M. (2025). Emerging Trends in the Pharmacological and Therapeutic Potential of Ginger: From Traditional Medicine to Nanotechnological Innovations. Curr. Pharm. Des..

[66] Yousefi M., Mohammadi V.G., Shadnoush M., Khorshidian N., Mortazavian A.M. (2022). *Zingiber officinale* Essential Oil-Loaded Chitosan-Tripolyphosphate Nanoparticles: Fabrication, Characterization and In-Vitro Antioxidant and Antibacterial Activities. Food Sci. Technol. Int..

[67] Ahmad N., Ahmad R., Amir M., Alam M.A., Almakhamel M.Z., Ali A., Ahmad A., Ashraf K. (2021). Ischemic Brain Treated with 6-Gingerol Loaded Mucoadhesive Nanoemulsion via Intranasal Delivery and Their Comparative Pharmacokinetic Effect in Brain. J. Drug Deliv. Sci. Technol..

[68] Deleanu M., Toma L., Sanda G.M., Barbălată T., Niculescu L.Ş., Sima A.V., Deleanu C., Săcărescu L., Suciu A., Alexandru G. (2023). Formulation of Phytosomes with Extracts of Ginger Rhizomes and Rosehips with Improved Bioavailability, Antioxidant and Anti-Inflammatory Effects In Vivo. Pharmaceutics.

[69] Maleki H., Azadi H., Yousefpoor Y., Doostan M., Doostan M., Farzaei M.H. (2023). Encapsulation of Ginger Extract in Nanoemulsions: Preparation, Characterization and in Vivo Evaluation in Rheumatoid Arthritis. J. Pharm. Sci..

[70] Xie Q., Gu J., Sun Y., Hong J., Wang J., Li N., Zhang Y., Liu M., Zhang X., Zhao X. (2024). Therapeutic Potential of Ginger Exosome-Like Nanoparticles for Alleviating Periodontitis-Induced Tissue Damage. Int. J. Nanomed..

[71] Alshaikh F., Al-Samydai A., Issa R., Alshaer W., Alqaraleh M., Al-Halaseh L.K., Alsanabrah A., Ghanim B.Y., Al Azzam K.M., Qinna N.A. (2024). Encapsulation of Gingerol into Nanoliposomes: Evaluation of In Vitro Anti-Inflammatory and Anti-Cancer Activity. Biomed. Chromatogr. BMC.

[72] Zhang M., Viennois E., Prasad M., Zhang Y., Wang L., Zhang Z., Han M.K., Xiao B., Xu C., Srinivasan S. (2016). Edible Ginger-Derived Nanoparticles: A Novel Therapeutic Approach for the Prevention and Treatment of Inflammatory Bowel Disease and Colitis-Associated Cancer. Biomaterials.

[73] Plengsuriyakarn T., Na-Bangchang K. (2020). Preclinical Toxicology and Anticholangiocarcinoma Activity of Oral Formulation of Standardized Extract of *Zingiber officinale*. Planta Med..

[74] Qiao Z., Zhang K., Liu J., Cheng D., Yu B., Zhao N., Xu F.-J. (2022). Biomimetic Electrodynamic Nanoparticles Comprising Ginger-Derived Extracellular Vesicles for Synergistic Anti-Infective Therapy. Nat. Commun..

[75] Abd El Wahab W.M., El-Badry A.A., Mahmoud S.S., El-Badry Y.A., El-Badry M.A., Hamdy D.A. (2021). Ginger (*Zingiber officinale*)-Derived Nanoparticles in *Schistosoma mansoni* Infected Mice: Hepatoprotective and Enhancer of Etiological Treatment. PLoS Negl. Trop. Dis..

[76] Olvera Lopez E., Ballard B.D., Jan A. (2025). Cardiovascular Disease. StatPearls.

[77] Nayebifar S., Afzalpour M.E., Kazemi T., Eivary S.H.A., Mogharnasi M. (2016). The Effect of a 10-Week High-Intensity Interval Training and Ginger Consumption on Inflammatory Indices Contributing to Atherosclerosis in Overweight Women. J. Res. Med. Sci..

[78] Hanifah N., Achmad Y.F., Humaira A., Salasia S.I.O. (2021). Red Ginger-Extract Nanoemulsion Modulates High Blood Pressure in Rats by Regulating Angiotensin-Converting Enzyme Production. Vet. World.

[79] Arablou T., Aryaeian N., Valizadeh M., Sharifi F., Hosseini A., Djalali M. (2014). The Effect of Ginger Consumption on Glycemic Status, Lipid Profile and Some Inflammatory Markers in Patients with Type 2 Diabetes Mellitus. Int. J. Food Sci. Nutr..

[80] Makhdoomi Arzati M., Mohammadzadeh Honarvar N., Saedisomeolia A., Anvari S., Effatpanah M., Makhdoomi Arzati R., Yekaninejad M.S., Hashemi R., Djalali M. (2017). The Effects of Ginger on Fasting Blood Sugar, Hemoglobin A1c, and Lipid Profiles in Patients with Type 2 Diabetes. Int. J. Endocrinol. Metab..

[81] Azimi P., Ghiasvand R., Feizi A., Hosseinzadeh J., Bahreynian M., Hariri M., Khosravi-Boroujeni H. (2016). Effect of Cinnamon, Cardamom, Saffron and Ginger Consumption on Blood Pressure and a Marker of Endothelial Function in Patients with Type 2 Diabetes Mellitus: A Randomized Controlled Clinical Trial. Blood Press..

[82] Maharlouei N., Tabrizi R., Lankarani K.B., Rezaianzadeh A., Akbari M., Kolahdooz F., Rahimi M., Keneshlou F., Asemi Z. (2019). The Effects of Ginger Intake on Weight Loss and Metabolic Profiles among Overweight and Obese Subjects: A Systematic Review and Meta-Analysis of Randomized Controlled Trials. Crit. Rev. Food Sci. Nutr..

[83] Hasani H., Arab A., Hadi A., Pourmasoumi M., Ghavami A., Miraghajani M. (2019). Does Ginger Supplementation Lower Blood Pressure? A Systematic Review and Meta-Analysis of Clinical Trials. Phytother. Res. PTR.

[84] Ghayur M.N., Gilani A.H. (2005). Ginger Lowers Blood Pressure through Blockade of Voltage-Dependent Calcium Channels. J. Cardiovasc. Pharmacol..

[85] Ghayur M.N., Gilani A.H., Afridi M.B., Houghton P.J. (2005). Cardiovascular Effects of Ginger Aqueous Extract and Its Phenolic Constituents Are Mediated through Multiple Pathways. Vascul. Pharmacol..

[86] Akinyemi A.J., Ademiluyi A.O., Oboh G. (2014). Inhibition of Angiotensin-1-Converting Enzyme Activity by Two Varieties of Ginger (*Zingiber officinale*) in Rats Fed a High Cholesterol Diet. J. Med. Food.

[87] Ji K., Fang L., Zhao H., Li Q., Shi Y., Xu C., Wang Y., Du L., Wang J., Liu Q. (2017). Ginger Oleoresin Alleviated γ-Ray Irradiation-Induced Reactive Oxygen Species via the Nrf2 Protective Response in Human Mesenchymal Stem Cells. Oxid. Med. Cell. Longev..

[88] Ganjikunta V.S., Maddula R.R., Bhasha S., Sahukari R., Kondeti Ramudu S., Chenji V., Kesireddy S.R., Zheng Z., Korivi M. (2022). Cardioprotective Effects of 6-Gingerol against Alcohol-Induced ROS-Mediated Tissue Injury and Apoptosis in Rats. Molecules.

[89] Wu S., Zhu J., Wu G., Hu Z., Ying P., Bao Z., Ding Z., Tan X. (2022). 6-Gingerol Alleviates Ferroptosis and Inflammation of Diabetic Cardiomyopathy via the Nrf2/HO-1 Pathway. Oxid. Med. Cell. Longev..

[90] Poprac P., Jomova K., Simunkova M., Kollar V., Rhodes C.J., Valko M. (2017). Targeting Free Radicals in Oxidative Stress-Related Human Diseases. Trends Pharmacol. Sci..

[91] Masuda Y., Kikuzaki H., Hisamoto M., Nakatani N. (2004). Antioxidant Properties of Gingerol Related Compounds from Ginger. BioFactors.

[92] Srinivasan K. (2017). Ginger Rhizomes (*Zingiber officinale*): A Spice with Multiple Health Beneficial Potentials. PharmaNutrition.

[93] Wu H.-C., Horng C.-T., Tsai S.-C., Lee Y.-L., Hsu S.-C., Tsai Y.-J., Tsai F.-J., Chiang J.-H., Kuo D.-H., Yang J.-S. (2018). Relaxant and Vasoprotective Effects of Ginger Extracts on Porcine Coronary Arteries. Int. J. Mol. Med..

[94] Abdi T., Mahmoudabady M., Marzouni H.Z., Niazmand S., Khazaei M. (2021). Ginger (*Zingiber officinale* Roscoe) Extract Protects the Heart Against Inflammation and Fibrosis in Diabetic Rats. Can. J. Diabetes.

[95] Yue Y., Meng K., Pu Y., Zhang X. (2017). Transforming Growth Factor Beta (TGF-β) Mediates Cardiac Fibrosis and Induces Diabetic Cardiomyopathy. Diabetes Res. Clin. Pract..

[96] El-Borm H.T., Gobara M.S., Badawy G.M. (2021). Ginger Extract Attenuates Labetalol Induced Apoptosis, DNA Damage, Histological and Ultrastructural Changes in the Heart of Rat Fetuses. Saudi J. Biol. Sci..

[97] Amran A.Z., Jantan I., Dianita R., Buang F. (2015). Protective Effects of the Standardized Extract of *Zingiber officinale* on Myocardium against Isoproterenol-Induced Biochemical and Histopathological Alterations in Rats. Pharm. Biol..

[98] El-Hawwary A.A., Omar N.M. (2019). The Influence of Ginger Administration on Cisplatin-Induced Cardiotoxicity in Rat: Light and Electron Microscopic Study. Acta Histochem..

[99] Subbaiah G.V., Mallikarjuna K., Shanmugam B., Ravi S., Taj P.U., Reddy K.S. (2017). Ginger Treatment Ameliorates Alcohol-Induced Myocardial Damage by Suppression of Hyperlipidemia and Cardiac Biomarkers in Rats. Pharmacogn. Mag..

[100] Ma S.-Q., Guo Z., Liu F.-Y., Hasan S.-G., Yang D., Tang N., An P., Wang M.-Y., Wu H.-M., Yang Z. (2021). 6-Gingerol Protects against Cardiac Remodeling by Inhibiting the P38 Mitogen-Activated Protein Kinase Pathway. Acta Pharmacol. Sin..

[101] Xue Y., Zhang M., Zheng B., Zhang Y., Chu X., Liu Y., Li Z., Han X., Chu L. (2021). [8]-Gingerol Exerts Anti-Myocardial Ischemic Effects in Rats via Modulation of the MAPK Signaling Pathway and L-Type Ca^2+^ Channels. Pharmacol. Res. Perspect..

[102] Foglio E., Puddighinu G., Germani A., Russo M.A., Limana F. (2017). HMGB1 Inhibits Apoptosis Following MI and Induces Autophagy via mTORC1 Inhibition. J. Cell. Physiol..

[103] Zhang W., Liu X., Jiang Y., Wang N., Li F., Xin H. (2019). 6-Gingerol Attenuates Ischemia-Reperfusion-Induced Cell Apoptosis in Human AC16 Cardiomyocytes through HMGB2-JNK1/2-NF-κB Pathway. Evid.-Based Complement. Altern. Med. ECAM.

[104] Alolga R.N., Wang F., Zhang X., Li J., Tran L.-S.P., Yin X. (2022). Bioactive Compounds from the Zingiberaceae Family with Known Antioxidant Activities for Possible Therapeutic Uses. Antioxidants.

[105] Zeng G.-F., Zhang Z.-Y., Lu L., Xiao D.-Q., Zong S.-H., He J.-M. (2013). Protective Effects of Ginger Root Extract on Alzheimer Disease-Induced Behavioral Dysfunction in Rats. Rejuvenation Res..

[106] Priyadarshini S., Goyal K., R R., Gupta S., Roy A., Biswas R., Patra S., Chauhan P., Wadhwa K., Singh G. (2025). Polypharmacology and Neuroprotective Effects of Gingerol in Alzheimer’s Disease. Mol. Neurobiol..

[107] Pan Q., Liu P., Wan M. (2023). 6-Gingerol Attenuates Sepsis-Induced Acute Lung Injury by Suppressing NLRP3 Inflammasome through Nrf2 Activation. Folia Histochem. Cytobiol..

[108] Simon A., Darcsi A., Kéry Á., Riethmüller E. (2020). Blood-Brain Barrier Permeability Study of Ginger Constituents. J. Pharm. Biomed. Anal..

[109] Liu Y., Li D., Wang S., Peng Z., Tan Q., He Q., Wang J. (2023). 6-Gingerol Ameliorates Hepatic Steatosis, Inflammation and Oxidative Stress in High-Fat Diet-Fed Mice through Activating LKB1/AMPK Signaling. Int. J. Mol. Sci..

[110] Aloliqi A.A. (2022). Therapeutic Potential of 6-Gingerol in Prevention of Colon Cancer Induced by Azoxymethane through the Modulation of Antioxidant Potential and Inflammation. Curr. Issues Mol. Biol..

[111] Chen M., Lin E., Xiao R., Li Z., Liu B., Wang J. (2024). Structural Characteristic, Strong Antioxidant, and Anti-Gastric Cancer Investigations on an Oleoresin from Ginger (*Zingiber officinale* Var. Roscoe). Foods.

[112] Chen C., Chen X., Mo Q., Liu J., Yao X., Di X., Qin Z., He L., Yao Z. (2023). Cytochrome P450 Metabolism Studies of [6]-Gingerol, [8]-Gingerol, and [10]-Gingerol by Liver Microsomes of Humans and Different Species Combined with Expressed CYP Enzymes. RSC Adv..

[113] Schadich E., Hlaváč J., Volná T., Varanasi L., Hajdúch M., Džubák P. (2016). Effects of Ginger Phenylpropanoids and Quercetin on Nrf2-ARE Pathway in Human BJ Fibroblasts and HaCaT Keratinocytes. BioMed Res. Int..

[114] Waiwut P., Takomthong P., Anorach R., Lomaboot N., Daodee S., Chulikhit Y., Monthakantirat O., Khamphukdee C., Boonyarat C. (2025). Pharmacological Evaluation of a Traditional Thai Polyherbal Formula for Alzheimer’s Disease: Evidence from In Vitro and In Silico Studies. Int. J. Mol. Sci..

[115] Bebawy G., Ashour A.A., El-Moslemany R.M., El-Habashy S.E., Bakr B.A., El-Kamel A.H., Heikal L.A. (2025). Contact Lenses Eluting Atorvastatin-Loaded Ginger-Exosomes: Mechanistic Modulation of Oxidative and Inflammatory Pathways in Glaucoma Management. Int. J. Pharm..

[116] Huang J., Lai L., Su Y., Chen J., Li P., Du B. (2025). Probiotic-Fermented Ginger-Processed Gastrodia Elata BI. Ameliorates AlCl3-Induced Cognitive Dysfunction in an Alzheimer’s Disease Rat Model by Regulating the Gut Microbiota and CREB/BDNF Pathway. Food Res. Int..

[117] Rezazadeh-Shojaee F.-S., Ramazani E., Kasaian J., Tayarani-Najaran Z. (2022). Protective Effects of 6-Gingerol on 6-Hydroxydopamine-Induced Apoptosis in PC12 Cells through Modulation of SAPK/JNK and Survivin Activation. J. Biochem. Mol. Toxicol..

[118] Kim J.H., Kim J.S., Ju I.G., Huh E., Choi Y., Lee S., Cho J.-Y., Park B.Y., Oh M.S. (2024). Coadministration of 6-Shogaol and Levodopa Alleviates Parkinson’s Disease-Related Pathology in Mice. Biomol. Ther..

[119] Talebi M., İlgün S., Ebrahimi V., Talebi M., Farkhondeh T., Ebrahimi H., Samarghandian S. (2021). *Zingiber officinale* Ameliorates Alzheimer’s Disease and Cognitive Impairments: Lessons from Preclinical Studies. Biomed. Pharmacother..

[120] Cryan J.F., O’Riordan K.J., Sandhu K., Peterson V., Dinan T.G. (2020). The Gut Microbiome in Neurological Disorders. Lancet Neurol..

[121] Lim S., Moon M., Oh H., Kim H.G., Kim S.Y., Oh M.S. (2014). Ginger Improves Cognitive Function via NGF-Induced ERK/CREB Activation in the Hippocampus of the Mouse. J. Nutr. Biochem..

[122] Zeng G., Zong S., Zhang Z., Fu S., Li K., Fang Y., Lu L., Xiao D.-Q. (2015). The Role of 6-Gingerol on Inhibiting Amyloid β Protein-Induced Apoptosis in PC12 Cells. Rejuvenation Res..

[123] Pan Y., Li Z., Zhao X., Du Y., Zhang L., Lu Y., Yang L., Cao Y., Qiu J., Qian Y. (2024). Screening of Active Substances Regulating Alzheimer’s Disease in Ginger and Visualization of the Effectiveness on 6-Gingerol Pathway Targets. Foods.

[124] Zahedi E., Naseri F.M., Zamani E., Nikbakhtzadeh M., Rastegar T., Sanaeirad A., Sadr S.S. (2025). Ginger Extract Improves Cognitive Dysfunction via Modulation of Gut Microbiota-Derived Short-Chain Fatty Acids in D-Galactose/Ovariectomy-Induced Alzheimer-Like Disease. Mol. Neurobiol..

[125] Watari H., Shimada Y., Matsui M., Tohda C. (2019). Kihito, a Traditional Japanese Kampo Medicine, Improves Cognitive Function in Alzheimer’s Disease Patients. Evid. Based Complement. Alternat. Med..

[126] Saenghong N., Wattanathorn J., Muchimapura S., Tongun T., Piyavhatkul N., Banchonglikitkul C., Kajsongkram T. (2012). *Zingiber officinale* Improves Cognitive Function of the Middle-Aged Healthy Women. Evid.-Based Complement. Altern. Med. ECAM.

[127] Kim J.H., Yoon H.-J., Choi Y., Kim J.S., Ju I.G., Eo H., Lee S., Cho J.-Y., Park B.Y., Hong S.-P. (2025). 6-Shogaol, a Neuro-Nutraceutical Derived from Ginger, Alleviates Motor Symptoms and Depression-like Behaviors and Modulates the Release of Monoamine Neurotransmitters in Parkinson’s Disease Mice. Eur. J. Nutr..

[128] O’Neill C. (2019). Gut Microbes Metabolize Parkinson’s Disease Drug. Science.

[129] Cui W., Guo Z., Chen X., Yan R., Ma W., Yang X., Lin Y. (2024). Targeting Modulation of Intestinal Flora through Oral Route by an Antimicrobial Nucleic Acid-Loaded Exosome-like Nanovesicles to Improve Parkinson’s Disease. Sci. Bull..

[130] Boutitah-Benyaich I., Eixarch H., Villacieros-Álvarez J., Hervera A., Cobo-Calvo Á., Montalban X., Espejo C. (2025). Multiple Sclerosis: Molecular Pathogenesis and Therapeutic Intervention. Signal Transduct. Target. Ther..

[131] Han J.-J., Li X., Ye Z.-Q., Lu X.-Y., Yang T., Tian J., Wang Y.-Q., Zhu L., Wang Z.-Z., Zhang Y. (2019). Treatment with 6-Gingerol Regulates Dendritic Cell Activity and Ameliorates the Severity of Experimental Autoimmune Encephalomyelitis. Mol. Nutr. Food Res..

[132] Moradi V., Esfandiary E., Ghanadian M., Ghasemi N., Rashidi B. (2022). The Effect of *Zingiber officinale* Extract on Preventing Demyelination of Corpus Callosum in a Rat Model of Multiple Sclerosis. Iran. Biomed. J..

[133] Kamankesh F., Ganji A., Ghazavi A., Mosayebi G. (2023). The Anti-Inflammatory Effect of Ginger Extract on the Animal Model of Multiple Sclerosis. Iran. J. Immunol. IJI.

[134] Sapkota A., Park S.J., Choi J.W. (2019). Neuroprotective Effects of 6-Shogaol and Its Metabolite, 6-Paradol, in a Mouse Model of Multiple Sclerosis. Biomol. Ther..

[135] Foshati S., Poursadeghfard M., Heidari Z., Amani R. (2023). The Effects of Ginger Supplementation on Common Gastrointestinal Symptoms in Patients with Relapsing-Remitting Multiple Sclerosis: A Double-Blind Randomized Placebo-Controlled Trial. BMC Complement. Med. Ther..

[136] Piekarz J., Picheta N., Pobideł J., Daniłowska K., Gil-Kulik P. (2025). Phytotherapy as an Adjunct to the Treatment of Rheumatoid Arthritis—A Systematic Review of Clinical Trials. Phytomed. Int. J. Phytother. Phytopharm..

[137] Phan P.V., Sohrabi A., Polotsky A., Hungerford D.S., Lindmark L., Frondoza C.G. (2005). Ginger Extract Components Suppress Induction of Chemokine Expression in Human Synoviocytes. J. Altern. Complement. Med..

[138] Mao Y., Liu C., Liu D., Wei X., Tan X., Zhou J., Yu X., Liu M. (2023). In Vitro Inhibitory Effect of Zingerone on TNFα-Stimulated Fibroblast-like Synoviocytes. Vitr. Cell. Dev. Biol. Anim..

[139] Chen X., Shen J., Zhao J.-M., Guan J., Li W., Xie Q.-M., Zhao Y.-Q. (2020). Cedrol Attenuates Collagen-Induced Arthritis in Mice and Modulates the Inflammatory Response in LPS-Mediated Fibroblast-like Synoviocytes. Food Funct..

[140] Li N., Li X., Deng L., Yang H., Gong Z., Wang Q., Pan D., Zeng S., Chen J. (2023). 6-Shogaol Inhibits the Proliferation, Apoptosis, and Migration of Rheumatoid Arthritis Fibroblast-like Synoviocytes via the PI3K/AKT/NF-κB Pathway. Phytomed. Int. J. Phytother. Phytopharm..

[141] Hosseinzadeh A., Bahrampour Juybari K., Fatemi M.J., Kamarul T., Bagheri A., Tekiyehmaroof N., Sharifi A.M. (2017). Protective Effect of Ginger (*Zingiber officinale* Roscoe) Extract against Oxidative Stress and Mitochondrial Apoptosis Induced by Interleukin-1β in Cultured Chondrocytes. Cells Tissues Organs.

[142] Shen C.-L., Hong K.-J., Kim S.W. (2005). Comparative Effects of Ginger Root (*Zingiber officinale* Rosc.) on the Production of Inflammatory Mediators in Normal and Osteoarthrotic Sow Chondrocytes. J. Med. Food.

[143] Villalvilla A., da Silva J.A., Largo R., Gualillo O., Vieira P.C., Herrero-Beaumont G., Gómez R. (2014). 6-Shogaol Inhibits Chondrocytes’ Innate Immune Responses and Cathepsin-K Activity. Mol. Nutr. Food Res..

[144] Fouda A.M., Berika M.Y. (2009). Evaluation of the Effect of Hydroalcoholic Extract of *Zingiber officinale* Rhizomes in Rat Collagen-induced Arthritis. Basic Clin. Pharmacol. Toxicol..

[145] Hwang J.H., Jung H.W., Oh S.Y., Kang J.-S., Kim J.-P., Park Y.-K. (2017). Effects of *Zingiber officinale* Extract on Collagen-Induced Arthritis in Mice and IL-1β-Induced Inflammation in Human Synovial Fibroblasts. Eur. J. Inflamm..

[146] Liu C., Li Y., Wen C., Yan Z., Olatunji O.J., Yin Z. (2022). Dehydrozingerone Alleviates Hyperalgesia, Oxidative Stress and Inflammatory Factors in Complete Freund’s Adjuvant-Induced Arthritic Rats. Drug Des. Dev. Ther..

[147] Mozaffari-Khosravi H., Naderi Z., Dehghan A., Nadjarzadeh A., Fallah Huseini H. (2016). Effect of Ginger Supplementation on Proinflammatory Cytokines in Older Patients with Osteoarthritis: Outcomes of a Randomized Controlled Clinical Trial. J. Nutr. Gerontol. Geriatr..

[148] Bartels E.M., Folmer V.N., Bliddal H., Altman R.D., Juhl C., Tarp S., Zhang W., Christensen R. (2015). Efficacy and Safety of Ginger in Osteoarthritis Patients: A Meta-Analysis of Randomized Placebo-Controlled Trials. Osteoarthr. Cartil..

[149] Baek H.-I., Shen L., Ha K.-C., Park Y.K., Kim C.S., Kwon J.E., Park S.J. (2024). Effectiveness and Safety of Steamed Ginger Extract on Mild Osteoarthritis: A Randomized, Double-Blind, Placebo-Controlled Clinical Trial. Food Funct..

[150] Broeckel J., Estes L., Leonard M., Dickerson B., Gonzalez D.E., Purpura M., Jäger R., Sowinski R., Rasmussen C.J., Kreider R.B. (2025). Effects of Ginger Supplementation in Individuals with Mild to Moderate Joint Pain I: Ratings of Pain, Functional Capacity, and Markers of Inflammation. J. Int. Soc. Sports Nutr..

[151] Estes L., Broeckel J., Leonard M., Dickerson B., Gonzalez D.E., Purpura M., Jäger R., Sowinski R., Rasmussen C.J., Kreider R.B. (2025). Effects of Ginger Supplementation in Individuals with Mild-to-Moderate Joint Pain II: Joint Flexibility, Markers of Health, Quality of Life, Analgesic Use, and Side Effects. J. Int. Soc. Sports Nutr..

[152] Bhattacharya E., Pal U., Dutta R., Bhowmik P.C., Mandal Biswas S. (2022). Antioxidant, Antimicrobial and DNA Damage Protecting Potential of Hot Taste Spices: A Comparative Approach to Validate Their Utilization as Functional Foods. J. Food Sci. Technol..

[153] Huang R., Zhou P.-K. (2021). DNA Damage Repair: Historical Perspectives, Mechanistic Pathways and Clinical Translation for Targeted Cancer Therapy. Signal Transduct. Target. Ther..

[154] El-Sharaky A.S., Newairy A.A., Kamel M.A., Eweda S.M. (2009). Protective Effect of Ginger Extract against Bromobenzene-Induced Hepatotoxicity in Male Rats. Food Chem. Toxicol..

[155] Ahmed R.S., Suke S.G., Seth V., Chakraborti A., Tripathi A.K., Banerjee B.D. (2008). Protective Effects of Dietary Ginger (*Zingiber officinales* Rosc.) on Lindane-Induced Oxidative Stress in Rats. Phytother. Res. PTR.

[156] Danwilai K., Konmun J., Sripanidkulchai B.-O., Subongkot S. (2017). Antioxidant Activity of Ginger Extract as a Daily Supplement in Cancer Patients Receiving Adjuvant Chemotherapy: A Pilot Study. Cancer Manag. Res..

[157] Kay J., Thadhani E., Samson L., Engelward B. (2019). Inflammation-Induced DNA Damage, Mutations and Cancer. DNA Repair.

[158] Wali A.F., Rehman M.U., Raish M., Kazi M., Rao P.G.M., Alnemer O., Ahmad P., Ahmad A. (2020). Zingerone [4-(3-Methoxy-4-Hydroxyphenyl)-Butan-2] Attenuates Lipopolysaccharide-Induced Inflammation and Protects Rats from Sepsis Associated Multi Organ Damage. Molecules.

[159] Navarro-Ibarra M.J., Hernández J., Caire-Juvera G. (2019). Diet, Physical Activity and Telomere Length in Adults. Nutr. Hosp..

[160] Kumari R., Jat P. (2021). Mechanisms of Cellular Senescence: Cell Cycle Arrest and Senescence Associated Secretory Phenotype. Front. Cell Dev. Biol..

[161] McHugh D., Gil J. (2018). Senescence and Aging: Causes, Consequences, and Therapeutic Avenues. J. Cell Biol..

[162] Kuilman T., Michaloglou C., Mooi W.J., Peeper D.S. (2010). The Essence of Senescence. Genes Dev..

[163] Salama R., Sadaie M., Hoare M., Narita M. (2014). Cellular Senescence and Its Effector Programs. Genes Dev..

[164] van Deursen J.M. (2014). The Role of Senescent Cells in Ageing. Nature.

[165] Walston J.D. (2012). Sarcopenia in Older Adults. Curr. Opin. Rheumatol..

[166] Saxton R.A., Sabatini D.M. (2017). mTOR Signaling in Growth, Metabolism, and Disease. Cell.

[167] Cayo A., Segovia R., Venturini W., Moore-Carrasco R., Valenzuela C., Brown N. (2021). mTOR Activity and Autophagy in Senescent Cells, a Complex Partnership. Int. J. Mol. Sci..

[168] Ito K., Hirao A., Arai F., Takubo K., Matsuoka S., Miyamoto K., Ohmura M., Naka K., Hosokawa K., Ikeda Y. (2006). Reactive Oxygen Species Act through P38 MAPK to Limit the Lifespan of Hematopoietic Stem Cells. Nat. Med..

[169] Lee J.-M., Li J., Johnson D.A., Stein T.D., Kraft A.D., Calkins M.J., Jakel R.J., Johnson J.A. (2005). Nrf2, a Multi-Organ Protector?. FASEB J..

[170] Yusof W.Z. (1988). Gingival Hyperplasia: An Intra-Oral Side Effect of Phenytoin, Nifedipine and Cyclosporine Therapies. Singap. Med. J..

[171] Moaddel R., Rossi M., Rodriguez S., Munk R., Khadeer M., Abdelmohsen K., Gorospe M., Ferrucci L. (2022). Identification of Gingerenone A as a Novel Senolytic Compound. PLoS ONE.

[172] Sivapathasuntharam C., Sivaprasad S., Hogg C., Jeffery G. (2019). Improving Mitochondrial Function Significantly Reduces the Rate of Age Related Photoreceptor Loss. Exp. Eye Res..

[173] Theurey P., Pizzo P. (2018). The Aging Mitochondria. Genes.

[174] Wang R., Santos J.M., Dufour J.M., Stephens E.R., Miranda J.M., Washburn R.L., Hibler T., Kaur G., Lin D., Shen C.-L. (2022). Ginger Root Extract Improves GI Health in Diabetic Rats by Improving Intestinal Integrity and Mitochondrial Function. Nutrients.

[175] Li Y., Tran V.H., Kota B.P., Nammi S., Duke C.C., Roufogalis B.D. (2014). Preventative Effect of *Zingiber officinale* on Insulin Resistance in a High-Fat High-Carbohydrate Diet-Fed Rat Model and Its Mechanism of Action. Basic Clin. Pharmacol. Toxicol..

[176] Kwak H.-B. (2013). Exercise and Obesity-Induced Insulin Resistance in Skeletal Muscle. Integr. Med. Res..

[177] Monda A., de Stefano M.I., Villano I., Allocca S., Casillo M., Messina A., Monda V., Moscatelli F., Dipace A., Limone P. (2024). Ultra-Processed Food Intake and Increased Risk of Obesity: A Narrative Review. Foods.

[178] Safaei M., Sundararajan E.A., Driss M., Boulila W., Shapi’i A. (2021). A Systematic Literature Review on Obesity: Understanding the Causes & Consequences of Obesity and Reviewing Various Machine Learning Approaches Used to Predict Obesity. Comput. Biol. Med..

[179] Ling C., Rönn T. (2019). Epigenetics in Human Obesity and Type 2 Diabetes. Cell Metab..

[180] Feng J.X., Riddle N.C. (2020). Epigenetics and Genome Stability. Mamm. Genome.

[181] Kawase Y., Sunagawa Y., Shimizu K., Funamoto M., Hamabe-Horiike T., Katanasaka Y., Shimizu S., Hawke P., Mori K., Komiyama M. (2023). 6-Shogaol, an Active Component of Ginger, Inhibits P300 Histone Acetyltransferase Activity and Attenuates the Development of Pressure-Overload-Induced Heart Failure. Nutrients.

[182] Khazbak A.E.-R., Salem S., Rady H., Bashandy M. (2023). Regulation of Breast Cancer Stem Cells Associated-miRNAs by Ginger and Persimmon Extracts. Egypt. Acad. J. Biol. Sci. C Physiol. Mol. Biol..

[183] Zhang M., Zhao R., Wang D., Wang L., Zhang Q., Wei S., Lu F., Peng W., Wu C. (2021). Ginger (*Zingiber officinale* Rosc.) and Its Bioactive Components Are Potential Resources for Health Beneficial Agents. Phytother. Res. PTR.

[184] Baek H.-I., Ha N.-R., Kim C., Im T.J., Kim Y.Y., Hwang S.H., Bae J.W. (2025). Efficacy and Safety of Steamed Ginger Extract for Gastric Health: A Randomized, Double-Blind, Placebo-Controlled Multi-Center Clinical Trial. Food Funct..

[185] Aregawi L.G., Zoltan C. (2025). Evaluation of Adverse Effects and Tolerability of Dietary Ginger Supplementation in Patients with Functional Dyspepsia. Curr. Ther. Res. Clin. Exp..

[186] Bhargava R., Chasen M., Elten M., MacDonald N. (2020). The Effect of Ginger (*Zingiber officinale* Roscoe) in Patients with Advanced Cancer. Support. Care Cancer.

[187] Syed Z.A., Fahim A., Safdar M., Imtiaz R. (2024). Role of Ginger in Management of Nausea among Patients Receiving Chemotherapy. Pak. J. Med. Sci..

[188] Wang J., Chen Y., Hu X., Feng F., Cai L., Chen F. (2020). Assessing the Effects of Ginger Extract on Polyphenol Profiles and the Subsequent Impact on the Fecal Microbiota by Simulating Digestion and Fermentation In Vitro. Nutrients.

[189] Liu J.-P., Wang J., Zhou S.-X., Huang D.-C., Qi G.-H., Chen G.-T. (2022). Ginger Polysaccharides Enhance Intestinal Immunity by Modulating Gut Microbiota in Cyclophosphamide-Induced Immunosuppressed Mice. Int. J. Biol. Macromol..

[190] Bretto E., Ribaldone D.G., Caviglia G.P., Saracco G.M., Bugianesi E., Frara S. (2023). Inflammatory Bowel Disease: Emerging Therapies and Future Treatment Strategies. Biomedicines.

[191] Veauthier B., Hornecker J.R. (2018). Crohn’s Disease: Diagnosis and Management. Am. Fam. Physician.

[192] RaviKKumar V.R., Rathi S., Singh S., Patel B., Singh S., Chaturvedi K., Sharma B. (2023). A Comprehensive Review on Ulcer and Their Treatment. Chin. J. Appl. Physiol..

[193] Rashidian A., Mehrzadi S., Ghannadi A.R., Mahzooni P., Sadr S., Minaiyan M. (2014). Protective Effect of Ginger Volatile Oil against Acetic Acid-Induced Colitis in Rats: A Light Microscopic Evaluation. J. Integr. Med..

[194] Shin J.-K., Park J.H., Kim K.S., Kang T.H., Kim H.S. (2020). Antiulcer Activity of Steamed Ginger Extract against Ethanol/HCl-Induced Gastric Mucosal Injury in Rats. Molecules.

[195] Sistani Karampour N., Arzi A., Rezaie A., Pashmforoosh M., Kordi F. (2019). Gastroprotective Effect of Zingerone on Ethanol-Induced Gastric Ulcers in Rats. Medicina.

[196] Hao W., Chen Z., Yuan Q., Ma M., Gao C., Zhou Y., Zhou H., Wu X., Wu D., Farag M.A. (2022). Ginger Polysaccharides Relieve Ulcerative Colitis via Maintaining Intestinal Barrier Integrity and Gut Microbiota Modulation. Int. J. Biol. Macromol..

[197] Jing Y., Wang Z., Cheng W., Fan H., Zheng K., Zheng Y., Wu L. (2025). Structure Characterization and Treatment Effect of *Zingiber officinale* Polysaccharide on Dextran Sulfate Sodium-Induced Ulcerative Colitis. Foods.

[198] Kim H.-R., Noh E.-M., Kim S.-Y. (2023). Anti-Inflammatory Effect and Signaling Mechanism of 8-Shogaol and 10-Shogaol in a Dextran Sodium Sulfate-Induced Colitis Mouse Model. Heliyon.

[199] Yang Z., Yu J., Wong C.C. (2024). Gastrointestinal Cancer Patient Derived Organoids at the Frontier of Personalized Medicine and Drug Screening. Cells.

[200] Hu S.-M., Yao X.-H., Hao Y.-H., Pan A.-H., Zhou X.-W. (2020). 8-Gingerol Regulates Colorectal Cancer Cell Proliferation and Migration through the EGFR/STAT/ERK Pathway. Int. J. Oncol..

[201] Wang J.-H., Chen Y.-W., Hsieh S., Kung M.-L. (2025). Nanosized Ginger-Derived Phenolic Zingerone Obstructs Cell Cycle G2/M Progression and Initiates Apoptosis in Human Colorectal Cancer. Environ. Toxicol..

[202] Mansingh D.P., OJ S., Sali V.K., Vasanthi H.R. (2018). [6]-Gingerol-Induced Cell Cycle Arrest, Reactive Oxygen Species Generation, and Disruption of Mitochondrial Membrane Potential Are Associated with Apoptosis in Human Gastric Cancer (AGS) Cells. J. Biochem. Mol. Toxicol..

[203] Fayed A.M., Habeeb S.N., Samy W., Bassiouny K., Abd-El-Aziz A.A., AlKhafaf D.M.R., Shareef H.K., AbdElrahman M., Aldhalmi A.K., Obaida D.S. (2024). Anticancer Properties of Garlic and Ginger Extract in Colon Cancer Cell Line. Asian Pac. J. Cancer Prev. APJCP.

[204] Yavari M., Jaafari M.R., Mirzavi F., Mosayebi G., Ghazavi A., Ganji A. (2022). Anti-Tumor Effects of PEGylated-Nanoliposomes Containing Ginger Extract in Colorectal Cancer-Bearing Mice. Iran. J. Basic Med. Sci..

[205] Saptarini N.M., Sitorus E.Y., Levita J. (2013). Structure-Based in Silico Study of 6-Gingerol, 6-Ghogaol, and 6-Paradol, Active Compounds of Ginger (*Zingiber officinale*) as COX-2 Inhibitors. Int. J. Chem..

[206] Rampogu S., Baek A., Gajula R.G., Zeb A., Bavi R.S., Kumar R., Kim Y., Kwon Y.J., Lee K.W. (2018). Ginger (*Zingiber officinale*) Phytochemicals—Gingerenone-A and Shogaol Inhibit SaHPPK: Molecular Docking, Molecular Dynamics Simulations and In Vitro Approaches. Ann. Clin. Microbiol. Antimicrob..

[207] Park J., Beck B.R., Kim H.H., Lee S., Kang K. (2022). A Brief Review of Machine Learning-Based Bioactive Compound Research. Appl. Sci..

[208] Qi X., Zhao Y., Qi Z., Hou S., Chen J. (2024). Machine Learning Empowering Drug Discovery: Applications, Opportunities and Challenges. Molecules.

[209] Mamoudou H., Mune M.A.M. (2025). AI-Driven Bioactive Peptide Discovery of Next-Generation Metabolic Biotherapeutics. Appl. Food Res..

[210] Gollapalli P., Rao A.S.J., Manjunatha H., Selvan G.T., Shetty P., Kumari N.S. (2023). Systems Pharmacology and Pharmacokinetics Strategy to Decode Bioactive Ingredients and Molecular Mechanisms from *Zingiber officinale* as Phyto-Therapeutics against Neurological Diseases. Curr. Drug Discov. Technol..

[211] Vignesh A., Amal T.C., Sarvalingam A., Vasanth K. (2024). A Review on the Influence of Nutraceuticals and Functional Foods on Health. Food Chem. Adv..

[212] Naskar S., Sing D., Banerjee S., Shcherbakova A., Bandyopadhyay A., Kar A., Haldar P.K., Sharma N., Mukherjee P.K., Bandyopadhyay R. (2025). Rapid Quality Assessment and Traceability of Ginger Powder from Northeast India and Indian Market Based on near Infrared Spectroscopic Fingerprinting. Phytochem. Anal..

[213] Yang J.-S., Tsai S.-C., Hsu Y.-M., Bau D.-T., Tsai C.-W., Chang W.-S., Kuo S.-C., Kuo S.-C., Yu C.-C., Chiu Y.-J. (2024). Integrating Natural Product Research Laboratory with Artificial Intelligence: Advancements and Breakthroughs in Traditional Medicine. BioMedicine.

[214] Yang L., Wang H., Zhu Z., Yang Y., Xiong Y., Cui X., Liu Y. (2025). Network Pharmacology-Driven Sustainability: AI and Multi-Omics Synergy for Drug Discovery in Traditional Chinese Medicine. Pharmaceuticals.

[215] Li B., Tan K., Lao A.R., Wang H., Zheng H., Zhang L. (2024). A Comprehensive Review of Artificial Intelligence for Pharmacology Research. Front. Genet..

[216] Kant S., Deepika, Roy S. (2025). Artificial Intelligence in Drug Discovery and Development: Transforming Challenges into Opportunities. Discov. Pharm. Sci..

[217] Ko H., Kim B.S., Lee Y.E., Choi T.H., Lee Y., Youn H.-S., Gu G.J. (2024). Anti-Inflammatory Effects of Gingerenone A through Modulation of Toll-like Receptor Signaling Pathways. Eur. J. Pharmacol..

[218] Jiang X. (2025). Biological Mechanisms, Pharmacological and Pathological Activities, and Quality Optimization of Gingerols and Shogaols. J. Funct. Foods.

[219] Hong Y., Zhu S., Liu Y., Tian C., Xu H., Chen G., Tao L., Xie T. (2025). The Integration of Machine Learning into Traditional Chinese Medicine. J. Pharm. Anal..

[220] Zhang M.-M., Wang D., Lu F., Zhao R., Ye X., He L., Ai L., Wu C.-J. (2021). Identification of the Active Substances and Mechanisms of Ginger for the Treatment of Colon Cancer Based on Network Pharmacology and Molecular Docking. BioData Min..

[221] Rigby S.P. (2024). Uses of Molecular Docking Simulations in Elucidating Synergistic, Additive, and/or Multi-Target (SAM) Effects of Herbal Medicines. Molecules.

[222] Sardarpour N., Goodarzi Z., Gharaghani S. (2024). Docking-Based Virtual Screening Method to Selection of Natural Compounds with Synergistic Inhibitory Effects Against Cancer Signaling Pathways Using Multi-Target Approach. Iran. J. Biotechnol..

[223] Zhou X., Afzal S., Wohlmuth H., Münch G., Leach D., Low M., Li C.G. (2022). Synergistic Anti-Inflammatory Activity of Ginger and Turmeric Extracts in Inhibiting Lipopolysaccharide and Interferon-γ-Induced Proinflammatory Mediators. Molecules.

[224] Rudrapal M., Eltayeb W.A., Rakshit G., El-Arabey A.A., Khan J., Aldosari S.M., Alshehri B., Abdalla M. (2023). Dual Synergistic Inhibition of COX and LOX by Potential Chemicals from Indian Daily Spices Investigated through Detailed Computational Studies. Sci. Rep..

[225] Swetha M., Keerthana C.K., Rayginia T.P., Anto R.J. (2021). Cancer Chemoprevention: A Strategic Approach Using Phytochemicals. Front. Pharmacol..

[226] Koirala M., Yan L., Mohamed Z., DiPaola M. (2025). AI-Integrated QSAR Modeling for Enhanced Drug Discovery: From Classical Approaches to Deep Learning and Structural Insight. Int. J. Mol. Sci..

[227] Sankar J., Rajendran V., Kuriakose B.B., Alhazmi A.H., Wong L.S., Muthusamy K. (2025). ML Enhanced Bioactivity Prediction for Angiotensin II Receptor: A Potential Anti-Hypertensive Drug Target. Sci. Rep..

[228] Peng S., Yu S., Zhang J., Zhang J. (2023). 6-Shogaol as a Novel Thioredoxin Reductase Inhibitor Induces Oxidative-Stress-Mediated Apoptosis in HeLa Cells. Int. J. Mol. Sci..

[229] Andrei C., Zanfirescu A., Nițulescu G.M., Olaru O.T., Negreș S. (2023). Natural Active Ingredients and TRPV1 Modulation: Focus on Key Chemical Moieties Involved in Ligand–Target Interaction. Plants.

[230] Alamsyah R.M., Satari M.H., Pintauli S., Iskandar S. (2023). Molecular Docking Analysis of Ginger (*Zingiber officinale*) on Dopamine Compare to Bupropion as Smoking Cessation. Pharmacia.

[231] Cheng X., Ruan Y., Dai J., Fan H., Ling J., Chen J., Lu W., Gao X., Cao P. (2024). 8-Shogaol Derived from Dietary Ginger Alleviated Acute and Inflammatory Pain by Targeting TRPV1. Phytomedicine.

[232] Fajrin F.A., Nugroho A.E., Susilowati R., Nurrochmad A. (2018). Molecular Docking Analysis of Ginger Active Compound on Transient Receptor Potential Cation Channel Subfamily V Member 1 (TRPV1). Indones. J. Chem..

[233] Demirel H.C., Arici M.K., Tuncbag N. (2022). Computational Approaches Leveraging Integrated Connections of Multi-Omic Data toward Clinical Applications. Mol. Omics.

[234] Vitorino R. (2024). Transforming Clinical Research: The Power of High-Throughput Omics Integration. Proteomes.

[235] Bonmatí L.M., Miguel A., Suárez A., Aznar M., Beregi J.P., Fournier L., Neri E., Laghi A., França M., Sardanelli F. (2022). CHAIMELEON Project: Creation of a Pan-European Repository of Health Imaging Data for the Development of AI-Powered Cancer Management Tools. Front. Oncol..

[236] Qadri Y.A., Shaikh S., Ahmad K., Choi I., Kim S.W., Vasilakos A.V. (2025). Explainable Artificial Intelligence: A Perspective on Drug Discovery. Pharmaceutics.

[237] Ding Q., Yao R., Bai Y., Da L., Wang Y., Xiang R., Jiang X., Zhai F. (2025). Explainable Artificial Intelligence in the Field of Drug Research. Drug Des. Dev. Ther..

[238] Ho S.-C., Chang Y.-H. (2018). Comparison of Inhibitory Capacities of 6-, 8- and 10-Gingerols/Shogaols on the Canonical NLRP3 Inflammasome-Mediated IL-1β Secretion. Molecules.

[239] Hu Y., Liu T., Zheng G., Zhou L., Ma K., Xiong X., Zheng C., Li J., Zhu Y., Bian W. (2023). Mechanism Exploration of 6-Gingerol in the Treatment of Atherosclerosis Based on Network Pharmacology, Molecular Docking and Experimental Validation. Phytomedicine.

[240] Marzouk N.H., Selim S., Elattar M., Mabrouk M.S., Mysara M. (2025). A Comprehensive Landscape of AI Applications in Broad-Spectrum Drug Interaction Prediction: A Systematic Review. J. Cheminform..

